# Sildenafil-evoked photoreceptor oxidative stress *in vivo* is unrelated to impaired visual performance in mice

**DOI:** 10.1371/journal.pone.0245161

**Published:** 2021-03-04

**Authors:** Bruce A. Berkowitz, Robert H. Podolsky, Karen Lins Childers, Aicha Saadane, Timothy S. Kern, Robin Roberts, Hailey Olds, Joydip Joy, Collin Richards, Tilman Rosales, Michael Schneider, Brennan Schilling, Arthur Orchanian, Emma Graffice, Kenan Sinan, Haohua Qian, Lamis Harp

**Affiliations:** 1 Department of Ophthalmology, Visual and Anatomical Sciences, Wayne State University School of Medicine, Detroit, Michigan, United States of America; 2 Beaumont Research Institute, Beaumont Health, Royal Oak, Michigan, United States of America; 3 Gavin Herbert Eye Institute, University of California Irvine, Irvine, California, United States of America; 4 Veterans Administration Medical Center Research Service, Long Beach, California, United States of America; 5 Visual Function Core National Eye Institute, National Institutes of Health, Bethesda, Maryland, United States of America; University of Florida, UNITED STATES

## Abstract

**Purpose:**

The phosphodiesterase inhibitor sildenafil is a promising treatment for neurodegenerative disease, but it can cause oxidative stress in photoreceptors *ex vivo* and degrade visual performance in humans. Here, we test the hypotheses that in wildtype mice sildenafil causes i) wide-spread photoreceptor oxidative stress *in vivo* that is linked with ii) impaired vision.

**Methods:**

In dark or light-adapted C57BL/6 mice ± sildenafil treatment, the presence of oxidative stress was evaluated in retina laminae *in vivo* by QUEnch-assiSTed (QUEST) magnetic resonance imaging, in the subretinal space *in vivo* by QUEST optical coherence tomography, and in freshly excised retina by a dichlorofluorescein assay. Visual performance indices were also evaluated by QUEST optokinetic tracking.

**Results:**

In light-adapted mice, 1 hr post-sildenafil administration, oxidative stress was most evident in the superior peripheral outer retina on both *in vivo* and *ex vivo* examinations; little evidence was noted for central retina oxidative stress *in vivo* and *ex vivo*. In dark-adapted mice 1 hr after sildenafil, no evidence for outer retina oxidative stress was found *in vivo*. Evidence for sildenafil-induced central retina rod cGMP accumulation was suggested as a panretinally thinner, dark-like subretinal space thickness in light-adapted mice at 1 hr but not 5 hr post-sildenafil. Cone-based visual performance was impaired by 5 hr post-sildenafil and not corrected with anti-oxidants; vision was normal at 1 hr and 24 hr post-sildenafil.

**Conclusions:**

The sildenafil-induced spatiotemporal pattern of oxidative stress in photoreceptors dominated by rods was unrelated to impairment of cone-based visual performance in wildtype mice.

## Introduction

Sildenafil, an inhibitor of phosphodiesterase type 5 (PDE 5, the enzyme responsible for cGMP hydrolysis), is useful for the treatment of erectile dysfunction and pulmonary arterial hypertension, and has been suggested as a potential therapeutic in neurodegenerative diseases including age-related macular degeneration and Alzheimer’s disease [1–4]. However, benefits from sildenafil therapy may be limited by its visual side effects that can range from mild to severe. For example, sildenafil can temporarily affect visual performance (although more persistent responses have been reported), and most electrophysiology studies in healthy retina have demonstrated a short-term negative impact [3, 5–10]. Of greater concern, sildenafil usage has been linked to idiopathic serous macular detachment [11]. In addition, sildenafil may increase the risk of neurodegeneration in, for example, the 1 in 50 people who are potential carriers of sight-threatening genetic defects underlying retinitis pigmentosa [12, 13]. Thus, a better understanding of the consequences of sildenafil on the visual system is needed to mitigate its side effects.

The above side effects in retina are commonly thought to arise from inhibition of PDE 5 located in cells of the inner retina and in the retinal pigment epithelium, as well as from inhibition of PDE 6 found in photoreceptors [14, 15]. One hypothesized consequence of such inhibition is a greater-than-normal cGMP content causing cyclic nucleotide-gated channels to remain open even in the light, resulting in widespread oxidative stress via increased intracellular calcium levels and ion pumping [16–19]. In photoreceptor cells *ex vivo*, phosphodiesterase inhibition causes excessive production of free radicals (i.e., oxidative stress), but it is unclear if this happens *in vivo* [17, 18]. Photoreceptor oxidative stress *per se* can impair visual performance by an unclear mechanism that may include acidification of the subretinal space [20–27].

In this study, we tested if sildenafil evokes panretinal oxidative stress in healthy retina *in vivo* and whether an induced oxidative stress is linked with impaired visual performance. Oxidative stress is non-invasively evaluated in retinal laminae using QUEnch-assiSTed (QUEST) magnetic resonance imaging (MRI), in subretinal space using QUEST optical coherence tomography (OCT), and in cone-based vision with QUEST optokinetic tracking (OKT) [20, 24, 28, 29]. QUEST MRI accurately evaluates panretinal, layer-specific, paramagnetic free radical-generated contrast as a decrease in 1/T1 with anti-oxidants [29, 30]. QUEST MRI oxidative stress measurements have been validated in animal models against several *ex vivo* assays [29]. In addition, we have shown positive QUEST MRI controls for localized oxidative stress only in certain parts of the retina, including treatment with either sodium iodate or diltiazem, or mutant mice with cre-dependent retinal pigment epithelium (RPE)-specific MnSOD knockout mice or PDE6b damage (rd10 mice) [20, 28, 31–33]. QUEST OCT tests whether anti-oxidants increase the light-adapted external limiting membrane to retinal pigment epithelium (ELM-RPE) thickness in central retina which is suppressed by oxidative stress, perhaps via induced acidosis [24–27]. The *in vivo* results were compared to conventional dichlorofluorescein (DCF) fluorescent maps of steady-state levels of reactive oxygen species in freshly excised retina [34]. Finally, QUEST OKT was used as a validated test of whether oxidative stress impaired cone-based visual performance [20]. Control mice were evaluated in groups given either sildenafil + saline, or sildenafil + an anti-oxidant drug combination that reduces excessive production of reactive oxygen species [i.e., methylene blue (MB) and α-lipoic acid (ALA)]. MB is an alternate electron transporter that effectively suppresses generation of superoxide from a variety of sources; ALA is a potent free radical neutralizer; both are FDA approved and are useful for QUEST MRI, OCT, and OKT studies [20, 24, 29, 35, 36].

## Materials and methods

All mice were treated in accordance with the National Institutes of Health Guide for the Care and Use of Laboratory Animals, the Association for Research in Vision and Ophthalmology Statement for the Use of Animals in Ophthalmic and Vision Research, and with specific authorization by the Wayne State University Division of Laboratory Animal Resources (DLAR) Institutional Animal and Care Use Committee (IACUC), and by National Eye Institute Animal Care and Use Committee. 2 mo male C57BL/6J mice (Jackson Laboratories, ME) were housed and maintained in full dark conditions or 12 hr:12 hr light-dark cycle laboratory lighting. Mice were humanely euthanized by an overdose of urethane followed by a cervical dislocation, as detailed in our IACUC-approved protocol. Data were collected from the left eye except for the OKT examination which used both eye’s.

### QUEST MRI

QUEST MRI was performed as previously described [20, 28, 29, 32, 33]. Mice were maintained in darkness for at least 16 hrs before all MRI studies. High-resolution MRI data were acquired on a 7T system (Bruker ClinScan, Billerica, MA) using a receive-only surface coil (1.0 cm diameter) centered on the left eye. One group of mice were kept in the dark (“dark”) throughout the preparation and MRI examination. For the light study, another group of mice (“light”) were exposed to room light (~ 300 lx) for 15 mins to 5 hours. In all groups, immediately before the MRI experiment, animals were anesthetized with urethane (36% solution intraperitoneally; 0.083 mL / 20 g animal weight, prepared fresh daily; Sigma–Aldrich, St. Louis, MO, USA) and treated topically with 1% atropine to ensure dilation of the iris during light exposure followed by 3.5% lidocaine gel to reduce eye motion. MRI data were acquired using several single spin-echo sequences (time to echo 13 ms, 7 × 7 mm^2^, matrix size 160 × 320, slice thickness 600 μm). Images were acquired at different repetition times (TR) in the following order (number per time between repetitions in parentheses): TR 0.15 seconds (6), 3.50 seconds (1), 1.00 seconds (2), 1.90 seconds (1), 0.35 seconds (4), 2.70 seconds (1), 0.25 seconds (5), and 0.50 seconds (3). To compensate for reduced signal–noise ratios at shorter TRs, progressively more images were collected as the TR decreased. The present resolution in the central retina is sufficient for extracting meaningful layer-specific anatomical and functional data, as previously discussed [37].

T1 data sets were collected from different groups of mice given an intraperitoneal injection of 29 mg/kg sildenafil, a dose that produces a transient impairment in retinal electrophysiology [12]. This dose is expected to produce a plasma level of sildenafil that is less than 2x that reported in humans [9]. In all cases, sildenafil was administered 45 min– 1 hr 15 min (i.e., ~1 hr) before QUEST MRI examination.

All mice were treated 24 hrs prior to study with 1 mg/kg MB (i.p., dissolved in saline) and then treated the next day with 29 mg/kg sildenafil ∼1 h before the MRI examination. 50 mg/kg ALA (i.p., dissolved in saline and pH adjusted to ∼7.4) was given 15–20 minutes post-sildenafil injection. Control mice were also given sildenafil as above but administered two saline injections rather than MB and ALA. The MB and ALA combination has been confirmed to suppress the excessive production of free radicals in several studies [20, 28, 32, 33].

### MRI data analysis

As previously described, for QUEST data, each T1 data set of 23 images was first processed by registering (rigid body; STACKREG plugin, ImageJ, Rasband, W.S., ImageJ, U. S. National Institutes of Health, Bethesda, Maryland, USA, https://imagej.nih.gov/ij/, 1997–2016) and then averaging images with the same TRs in order to generate a stack of 8 images [20, 28, 32, 33]. These averaged images were then registered (rigid body) across TRs. QUEST data were corrected for imperfect slice profile bias in the estimate of T1, as previously described (Chapter 18 in [38]). Briefly, by normalizing to the shorter TR, some of the bias can be reduced, giving a more precise estimate for T1. To achieve this normalization, we first apply a 3x3 Gaussian smoothing (performed three times) on only the TR 150 ms image to minimize noise and emphasize signal. The smoothed TR 150 ms image was then divided into the rest of the images in that T1 data set. Previously, we reported that this procedure helps to minimize day-to-day variation in the 1/T1 profile previously noted and obviated the need for a “vanilla control” group used previously for correcting for day-to-day variations [39, 40]. 1/T1 maps were calculated using the 7 normalized images via fitting to a three-parameter T1 equation (y = a + b*(exp(-c*TR)), where a, b, and c are fitted parameters) on a pixel-by-pixel basis using R (v.2.9.0, R Development Core Team [2009]). R: A language and environment for statistical computing. R Foundation for Statistical Computing, Vienna, Austria. ISBN 3–900051–07–0) scripts developed in-house, and the minpack.lm package (v.1.1.1, Timur V. Elzhov and Katharine M. Mullen minpack.lm: R interface to the Levenberg-Marquardt nonlinear least-squares algorithm found in MINPACK. R package version 1.1–1).

In each mouse, whole retinal thicknesses (μm) were objectively determined using the “half-height method” wherein a border is determined via a computer algorithm based on the crossing point at the midpoint between the local minimum and maximum, as detailed elsewhere [41, 42]. The distance between two neighboring crossing-points thus represents an objectively defined retinal thickness. 1/T1 profiles in each mouse were then normalized with 0% depth at the presumptive vitreoretinal border and 100% depth at the presumptive retina-choroid border. The present resolution is sufficient for extracting meaningful layer-specific anatomical and functional data, as previously discussed [43, 44].

**Fig 1:**
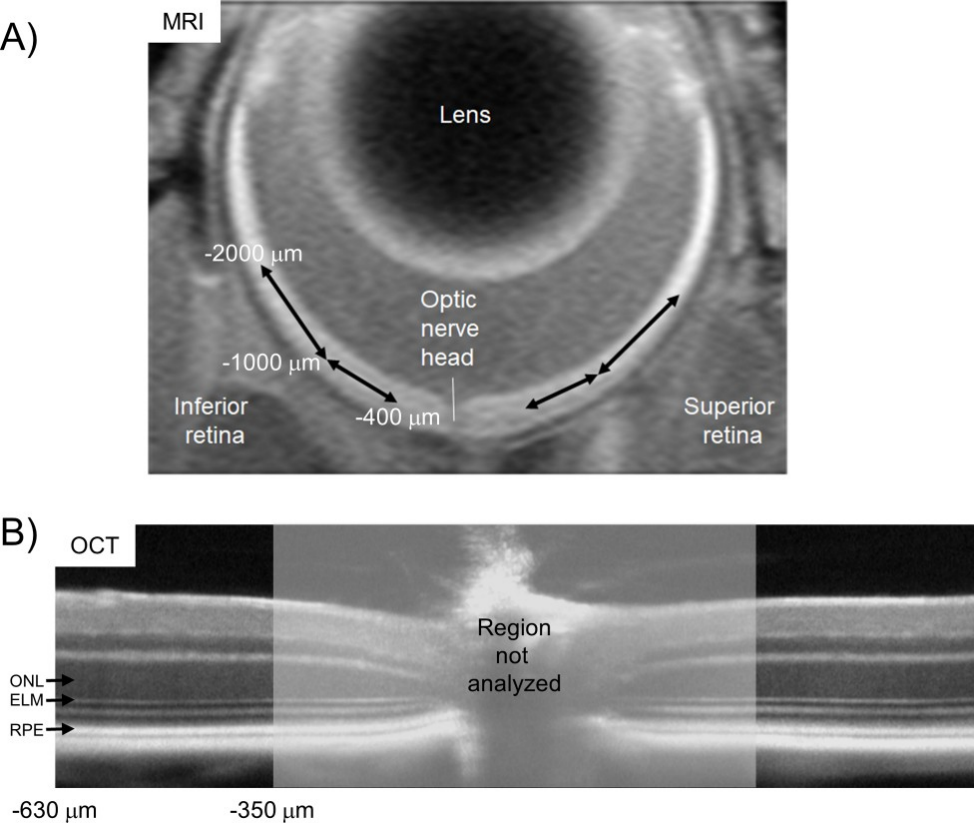
Comparison of MRI and OCT regions-of-interest (ROI’s). Four non-overlapping retinal regions were evaluated from MRI data A): ± 400–1000 μm and ± 1000–2000 μm from the optic nerve head for their transretinal 1/T1 profiles (shown in Figs 3 and 6). Two regions in central retina were studied from OCT data B): ± 350–630 μm from the optic nerve head to determine outer nuclear layer (ONL) thickness, and the external limiting membrane–retinal pigment epithelium (ELM-RPE) thickness.

We compared superior and inferior profiles separately from ± 400 to 1000 μm (central retina) and ± 1000 to 2000 μm (peripheral retina) from the optic nerve head (ONH) generated for each animal group (Fig 1). Excessive and asynchronous production of paramagnetic free radicals in retinal laminae is measured based on a reduction in 1/T1 with MB and ALA (i.e., a positive QUEST MRI response) [29]. However, an increase in 1/T1 in response to MB or ALA has no theoretical or biophysical basis linking it with oxidative stress. Thus, only significant decreases in 1/T1 following anti-oxidants are indicated on the graphs.

### DCF map *ex vivo* of steady-state levels of reactive oxygen species

The net result of production of reactive oxygen species (i.e., free radicals and H_2_O_2_) minus their elimination by endogenous antioxidants was mapped for each retina using a standard and well validated DCF assay [34, 45]. The interaction of the non-fluorescent probe 2’,7’-dichlorodihydrofluorescein (DCFH(2)) produces a fluorescent signal from its oxidation product, 2’,7’-dichlorofluorescein (DCF), that represents steady state levels of total reactive oxygen species [46]. DCF measurements were performed in subgroups of mice maintained in darkness for at least 16 hrs before euthanasia. The following day, light adaptation began for all mice at the same time; each mouse experienced between 30 min and 3 hour of room light. 1 hr before enucleation, mice (n = 3 / group) were given 29 mg/kg sildenafil or equal volume saline; dark-adapted mice were not examined since neither QUEST MRI nor QUEST OCT studies suggested oxidative stress in that group.

In all cases, eyes were enucleated and embedded in optimal cutting temperature compound and fresh frozen using liquid nitrogen vapor. DCF staining was conducted as previously reported [47]. Twelve microns sections were cut in vertical (inferior-superior) orientation, and while still frozen, sections were fixed in ice cold acetone at -80°C. Slides were then warmed to room temperature for at least 20 minutes. Section were washed in PBS 3 times in 5 minutes. Slides were transferred in humidified chamber, and sections were submerged in 10 μM DCF (D6883, Sigma-Aldrich Corp., St Louis, MO), and incubated at 37°C for 1 hour. Sections were then washed in PBS 3 times in 5 minutes, and one time in distilled water, cover-slipped using ProLong Gold anti-fade reagent with the nuclear stain 4′,6-diamidino-2-phenylindole (DAPI, P36935; Invitrogen, Carlsbad, CA, USA), and images taken by fluorescence microscopy (Keyence BZ-800 Series; Itasca, IL, USA) immediately after putting on the cover slip.

### QUEST optical coherence tomography (QUEST OCT)

**Fig 2:**
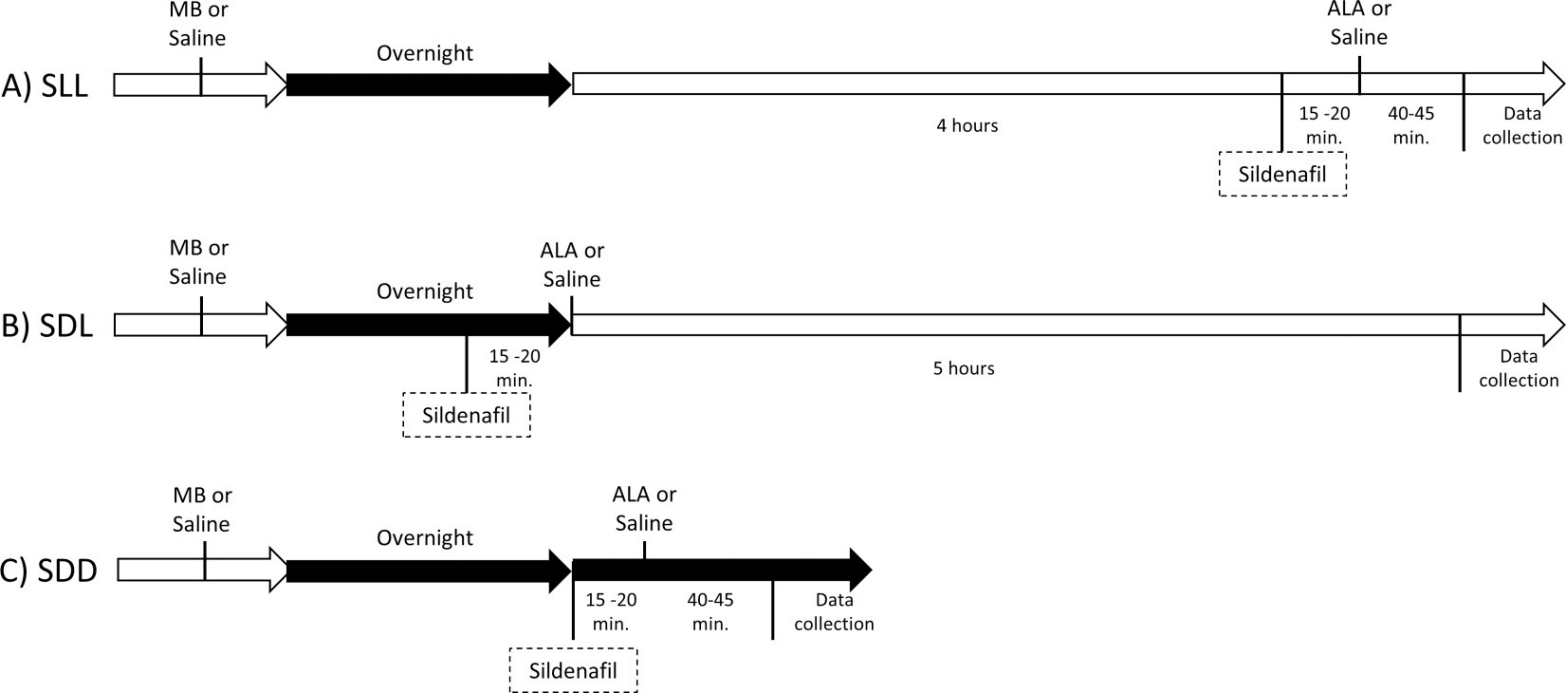
Timing diagrams for the three groups studied by OCT. A) Sildenafil is administered after 4 hr of room Light and studied 1 hr later in the Light (SLL). B) Sildenafil is given in the Dark then Light-adapted and studied 5 hr later (SDL). C) Sildenafil is given in the Dark and maintained as Dark-adapted during OCT examination (SDD). MB, methelyene blue; ALA; α-lipoic acid. Control groups were given saline.

Five groups were studied using QUEST OCT as previously described [24]. 1.) Light-adapted control mice: Age-matched C57BL/6 mice dark-adapted overnight and room light-adapted (~300 lx) for 5 hrs the following day, no injections beyond anesthesia. 2.) Dark-adapted control mice: Age-matched C57BL/6 mice dark-adapted overnight and studied in the dark the following day, no injections beyond anesthesia. For the remaining three groups, MB or saline are administered ~24 hr before the study and ALA or saline given 15–20 minutes after the sildenafil. 3.) For this group, Sildenafil is administered after 4 hr of room Light and studied 1 hr later in the Light (SLL, Fig 2A). 4.) For this group, Sildenafil is given in the Dark, then Light-adapted and studied 5 hr later (SDL, Fig 2B). 5.) For this group, Sildenafil is given in the Dark and studied 1 hr later in the Dark (SDD, Fig 2C).

In all groups, OCT (Envisu R2200 VHR SDOIS) was used to measure retinal layer spacing *in vivo*. Mice were anesthetized with urethane (36% solution intraperitoneally; 0.083 ml/20 g animal weight, prepared fresh weekly; Sigma-Aldrich, St. Louis, MO). 1% atropine sulfate was used to dilate the iris, and Systane Ultra was used to lubricate the eyes. Vertical B scan OCT was used in two ways. The first use is to identify retinal layers that contribute to the superior-inferior MRI profile data since aligning the vitreous-retina (0% depth) and retina-choroid (100% depth) borders of OCT and MRI images reasonably matches structure with function [25]. Second, separate groups of mice were studied in which the optic nerve is positioned to interrogate i) central, superior, and inferior retina (± 350–630 μm, Fig 1), ii) inferior retina (350–1191 μm), and iii) superior retina (350–1191 μm) [48]. The ELM-RPE and ONL thicknesses were measured using in-house developed R scripts that objectively identify layer boundaries after searching the space provided by a hand-drawn estimate (“seed boundaries”).

### QUEST optokinetic tracking (QUEST OKT)

As previously described, QUEST OKT was performed in separate experiments [20]. Four groups of mice were studied (only data collected before 12 pm are reported herein): 1.) Control group: Age-matched C57BL/6 mice, dark-adapted overnight and light-adapted for 5 hrs the following day, no injections beyond anesthesia. 2.) SLL group ± MB / ALA. 3.) SDL group ± MB / ALA. 4.) To see if visual performance recovers to baseline, we also examined a SLL 24 hr recovery group ± MB / ALA: In this group, light-adapted mice were given 29 mg/kg of sildenafil followed by 1 mg/kg MB or equal volume saline an hour later. Mice were dark-adapted overnight then given 50 mg/kg ALA or equal volume saline, brought into the light for 5 hours and studied by OKT.

For all groups, two cone-based visual performance metrics were evaluated in awake and freely moving mice using optokinetic tracking: spatial frequency thresholds (SFTs, “acuity”, in cycles/degree [c/d]) and contrast sensitivity (CS, measured near the peak of the nominal curve [0.06 cycles/degree [49]], inverse Michelson contrast [unitless]) (OptoMotry, CerebralMechanics Inc, Alberta, Canada). In brief, a vertical sine wave grating is projected as a virtual cylinder in 3-dimensional coordinate space on computer monitors arranged in a quadrangle around a testing arena. Unrestrained mice (as described above) are placed on an elevated platform at the center of the arena. An experimenter observed a video image of the platform from above to view the animal and follow the position of its head with the aid of a computer mouse and a crosshair continually placed on the mouse head as it moves. The X–Y positional coordinates of the crosshair are centered on the hub of the virtual cylinder, enabling its wall to be maintained at a constant “distance” from the animal’s eyes, and thereby fixing the spatial frequency of the stimulus at the animal’s viewing position. When the cylinder was rotated in the clockwise or counter-clockwise direction and the animal followed with head and neck movements that tracked the rotation, it was judged whether the animal’s visual system could distinguish the grating. Clockwise and counterclockwise tracking provide a measure of left and right eye SFT and CS [50]. After being in the light for 5 hrs, each set of SFT and peak of CS measurements per mouse can reliably be obtained in 30 minutes. Rod-based visual performance indices evaluated with OKT have limited dynamic range and were not considered to be useful for testing for impairment by oxidative stress [51].

### Statistical analysis

Data are presented as mean ± 95% confidence interval, and a significance level of 0.05 was used for all analyses. All outcomes (1/T1, OCT layer thickness, and OKT) had repeated measures for each mouse. As such, we used mixed models to analyze all outcomes using the Kenward-Roger method for calculating degrees of freedom in Proc Mixed and Proc Glimmix of SAS 9.4 (SAS software, Cary, NC, USA). For the MRI profile data (1/T1) and OCT layer thickness, we used cubic splines to model and compare mouse-specific profiles between groups. We used the same modeling strategy for both 1/T1 and OCT layer thickness. The number of “windows” (i.e., “knots”) with a relationship between outcome (1/T1, ELM-RPE thickness, or ONL thickness) and location (depth for MRI, distance from ONH for OCT layer thickness) was initially evaluated separately for each group for any given analysis, and the Akaike and Schwarz Bayesian information criteria (AIC and BIC) were used to identify the model with the fewest knots needed to model all groups. These initial models included full fixed effects (described separately for 1/T1 and OCT thickness below) and included a random intercept for each mouse nested within the appropriate treatment group. Additional random coefficients for side (indicator variable for superior side), region (indicator of 1000–2000 μm region), and location-specific coefficients (cubic spline coefficients) were evaluated using AIC and BIC. These models with the selected number of knots and random effects were then used to test for the fixed effects for each model, and non-significant interactions were removed to obtain the final model. The final model was then used to estimate mean profiles for all experimental conditions, and location-specific mean differences based on appropriate contrasts.

For 1/T1, each mouse was measured at 26 depths on two sides in two regions, resulting in 104 observations per mouse. We used Proc Mixed to fit the model for 1/T1, which included seven knots and the fixed effects for anti-oxidant (saline vs MB/ALA), side (inferior vs superior), condition (dark vs light), region (400–1000 μm vs 1000–2000 μm), as well as all interactions among these fixed effects and the locations. The final model included the random coefficients for side; region; linear relationship with depth; the fourth knot coefficient; all two-way interactions among side, region, and the fourth knot coefficient; and the interaction between region and the linear relationship with depth. The parameter estimates for the model are shown in S1 and S2 Tables in S5 File. The analysis of 1/T1 focused on identifying the depths at which an MB/ALA effect was statistically significant using pointwise hypothesis testing only when interactions were significant.

For the OCT data, each mouse was measured at 720 distances from the ONH on both sides of the ONH. We used Proc Glimmix to fit four models to the OCT data, a separate model for each outcome (changes in ELM-RPE or ONL) and for inferior and superior sides. All models included fixed effects for group (light-adapted control, CL; dark-adapted control, CD; SLL; SLL+MB/ALA; SDL; SDL+MB/ALA; SDD, SDD+MB/ALA), values for the spline coefficients for the distance from the ONH and the interaction between distance from the ONH and group. The number of knots and random coefficients used for the final models are shown in Table 1. The parameter estimates for these models are shown in S3–S10 Tables in S5 File. Contrasts were used with each final model to calculate a mean integrated across the entire layer. These contrasts were also used to compare the integrated means among the groups. We compared each group to the both controls and MB/ALA was compared only within a group (e.g., SLL vs SLL+MB/ALA). We used the Holm procedure to adjust for multiple comparisons for each focal group (SLL, SDL, and SDD).

**Table 1:** Model details for OCT layer thickness analysis.

Outcome	Position relative to ONH	Number of Knots	Random Coefficients
ELM-RPE thickness	Inferior	9	Intercept, Knot 7
	Superior	10	Intercept, Knot 7, Knot 8
ONL thickness	Inferior	7	Intercept, Knot 5
	Superior	9	Intercept, Knot 7

OKT was measured once per side, resulting in only two observations per mouse. As such, we used generalized linear mixed models (Proc Glimmix) to analyze both OKT measurements. For both acuity and contrast sensitivity, we included the fixed effect of group (saline, SLL, SLL+MB/ALA, SDL, SDL+MB/ALA, 24hr, 24hr+MB/ALA. Only a random intercept for mouse nested within group was included for these models. We used a normal distribution with the identity link for acuity and a gamma distribution with the log link for contrast sensitivity. The parameter estimates for the fit models are shown in S13–S16 Tables in S5 File. As with OCT distances, we compared each experimental group to saline, and compared the MB/ALA treatment within a focal group. We used the Holm procedure to adjust for multiple comparisons.

## Results

### Testing light-adapted mouse rods for oxidative stress 1 hr post-sildenafil (SLL)

#### QUEST MRI

**Fig 3:**
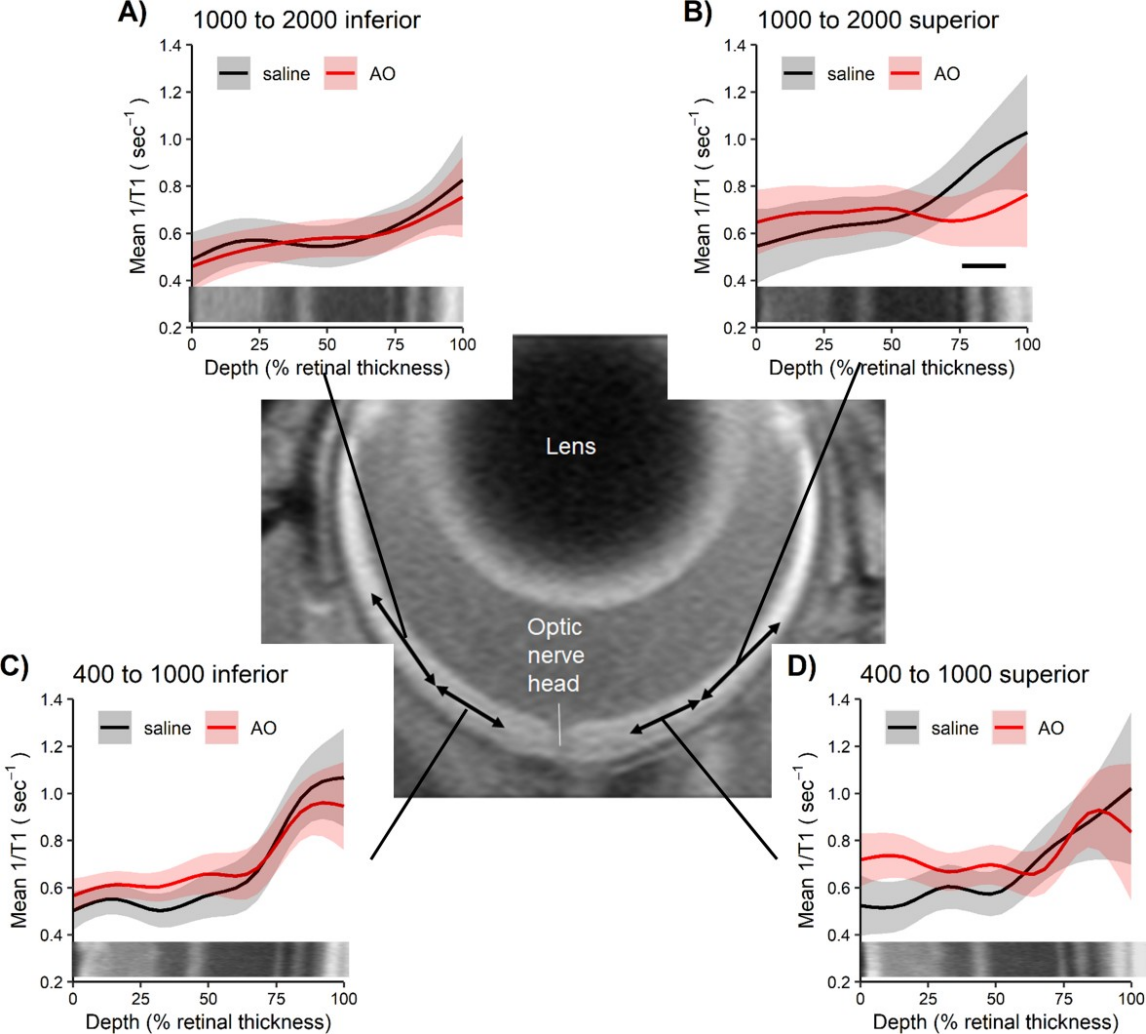
QUEST MRI showing oxidative stress localized to peripheral superior retina. Modeled 1/T1 profiles approximately 1 hour post-sildenafil IP in light-adapted mice given either saline (n = 4 mice, black line) or anti-oxidants (AO, n = 5 mice, red line) in these four retinal regions: A) 1000 to 2000 inferior, B) 1000 to 2000 superior, C) 400–1000 inferior, and D) 400–1000 superior; representative OCT images (bottom of each graph) provide spatial orientation. Only significant (horizontal black bar) AO reductions in 1/T1 indicative of oxidative stress are shown. Each profile has a solid line indicating the mean and a shaded region indicating 95% confidence intervals.

As shown in Fig 3 (see S1 Fig in S5 File for raw data), significant (p < 0.05) evidence for oxidative stress was found to be limited to the peripheral (i.e., 1000–2000 μm from the ONH) retina on the superior side (i.e., Fig 3B). Central (± 400–1000 μm) retina and peripheral inferior outer retina did not show evidence for oxidative stress (p > 0.05, Fig 3).

#### DCF

**Fig 4:**
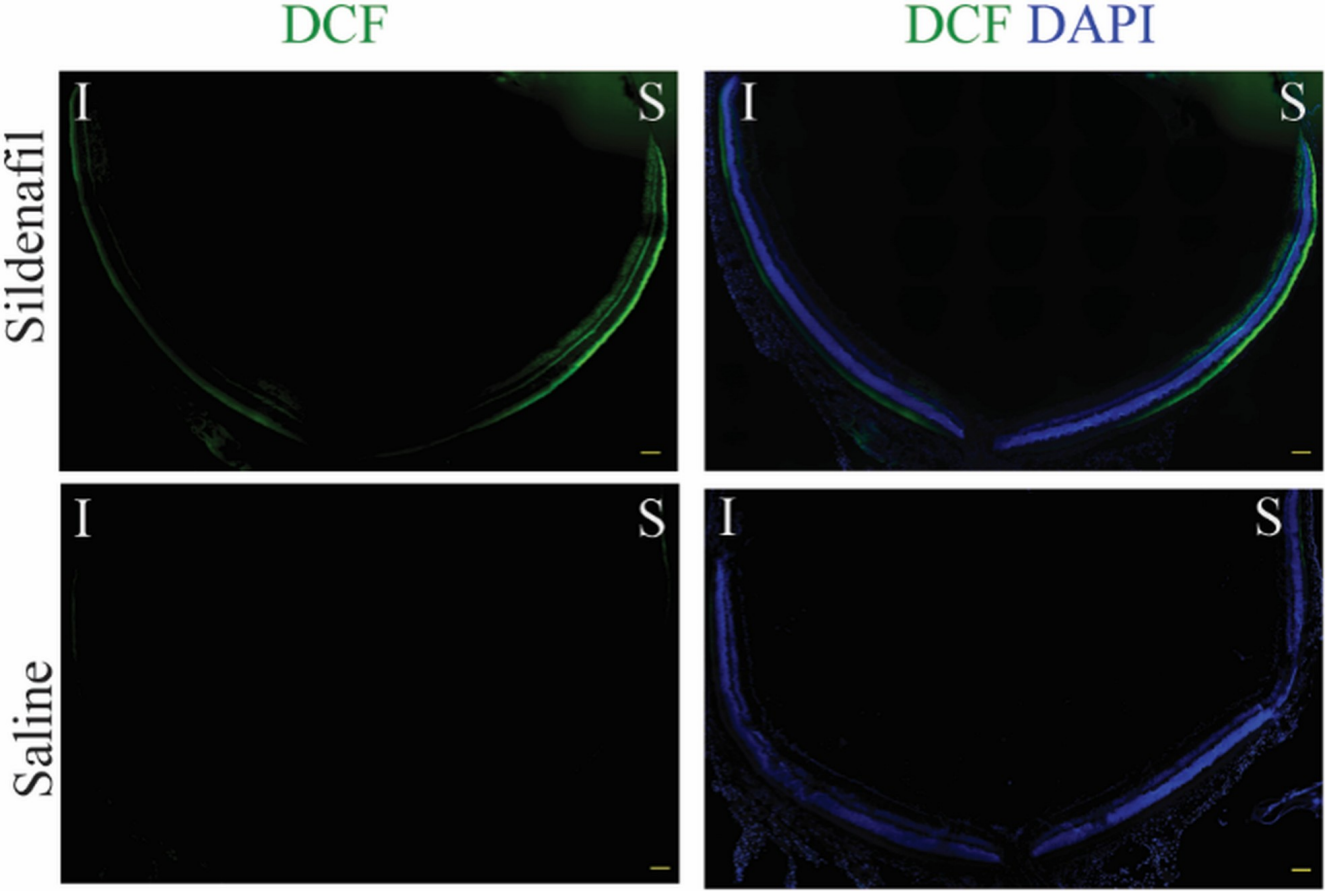
The effect of sildenafil on generation of oxidative stress in a representative *ex vivo* data set of unfixed cryosections stained with DCF. DCF staining for reactive oxygen species is shown in green; nuclei staining (DAPI) is shown in blue. The same intensity scaling is used in all images; scale bars are 100 μm.

A representative data set showing steady state levels of reactive oxygen species evaluated by DCF staining in freshly isolated retinal sections is shown in Fig 4. In saline treated mice, little DCF fluorescence was noted (“saline” row, Fig 4). In sildenafil treated mice, DCF signal was seen prominently in the superior peripheral outer retina (top row, Fig 4) with less signal observed in the inferior retina.

#### QUEST OCT

**Fig 5:**
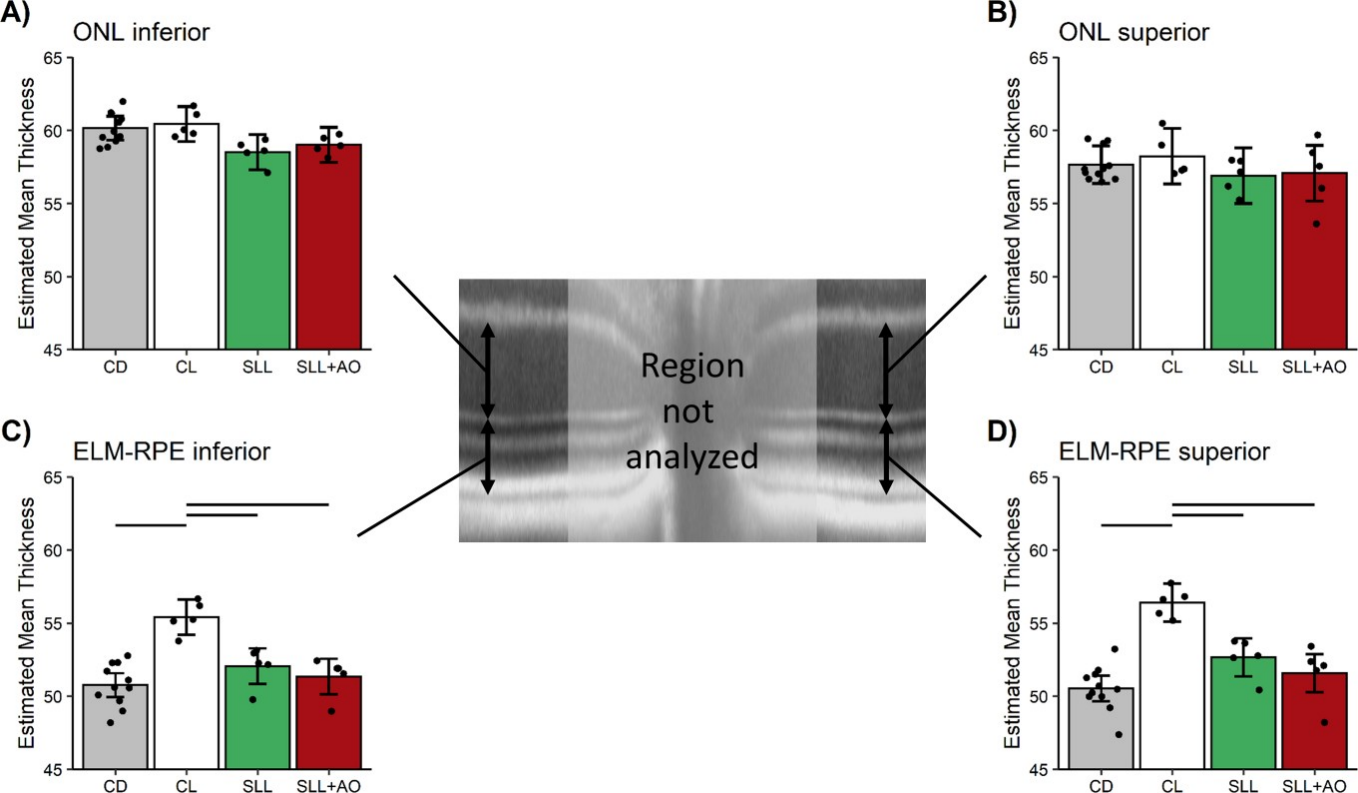
1 hr post-sildenafil after 4 hr of light-adaption mice (SLL, Fig 2) shows thinner, dark-like ELM-RPE. Modeled A) ONL inferior retina, B) ONL superior retina, C) ELM-RPE thickness inferior retina, and D) ELM-RPE thickness superior retina in uninjected control dark (CD, n = 11 mice, grey bar), control light (CL, n = 5 mice, white bar), SLL+saline (SLL, n = 5 mice, green bar), and SLL+AO (n = 5 mice, red bar) in the two different retinal regions. ONL is invariant to condition. ELM-RPE is significantly (horizontal black bar) thinner in the dark than in the light (CD vs. CL) as expected [44, 53, 54]. In SLL+saline and SLL+AO groups ELM-RPE thickness is not different from CD (and was thinner than CL); no evidence for oxidative stress was found. The points in each plot represent the estimated mean for each mouse based on the model. Error bars indicate 95% confidence intervals. Note the same control data sets are presented in each graph to facilitate comparisons.

In untreated control mice, superior and inferior central ELM-RPE was significantly (p < 0.05) ~5 μm thinner in the dark than after 5 hr of light-adaptation (Fig 5, and S3 Fig in S5 File for raw data), a similar effect size as previously reported using a higher spatial resolution OCT [52]. Light-dark data collected before 12:30 pm are reported herein. In the central retina (± 350–630 μm from the ONH), sildenafil reduced (p < 0.05) the ELM-RPE thickness from light- to dark-adapted levels in control mice (Fig 5); anti-oxidants did not (p > 0.05) restore ELM-RPE thickness to light-like values as would be expected if oxidative stress were present [24]. Similar results are found with a field-of-view ± 350–1191 μm from the optic nerve head (S4 Fig in S5 File).

In contrast to the ELM-RPE region, ONL was, as expected, invariant in dark and light-adapted control mice, and following administration of sildenafil (Fig 5 for ± 350–630 μm from the optic nerve head; ± 350–1191 μm from the optic nerve head data are shown in S4 Fig in S5 File) [44, 54].

### Testing light-adapted mouse for outer retina oxidative stress 5 hr post-sildenafil (SDL)

#### QUEST OCT

**Fig 6:**
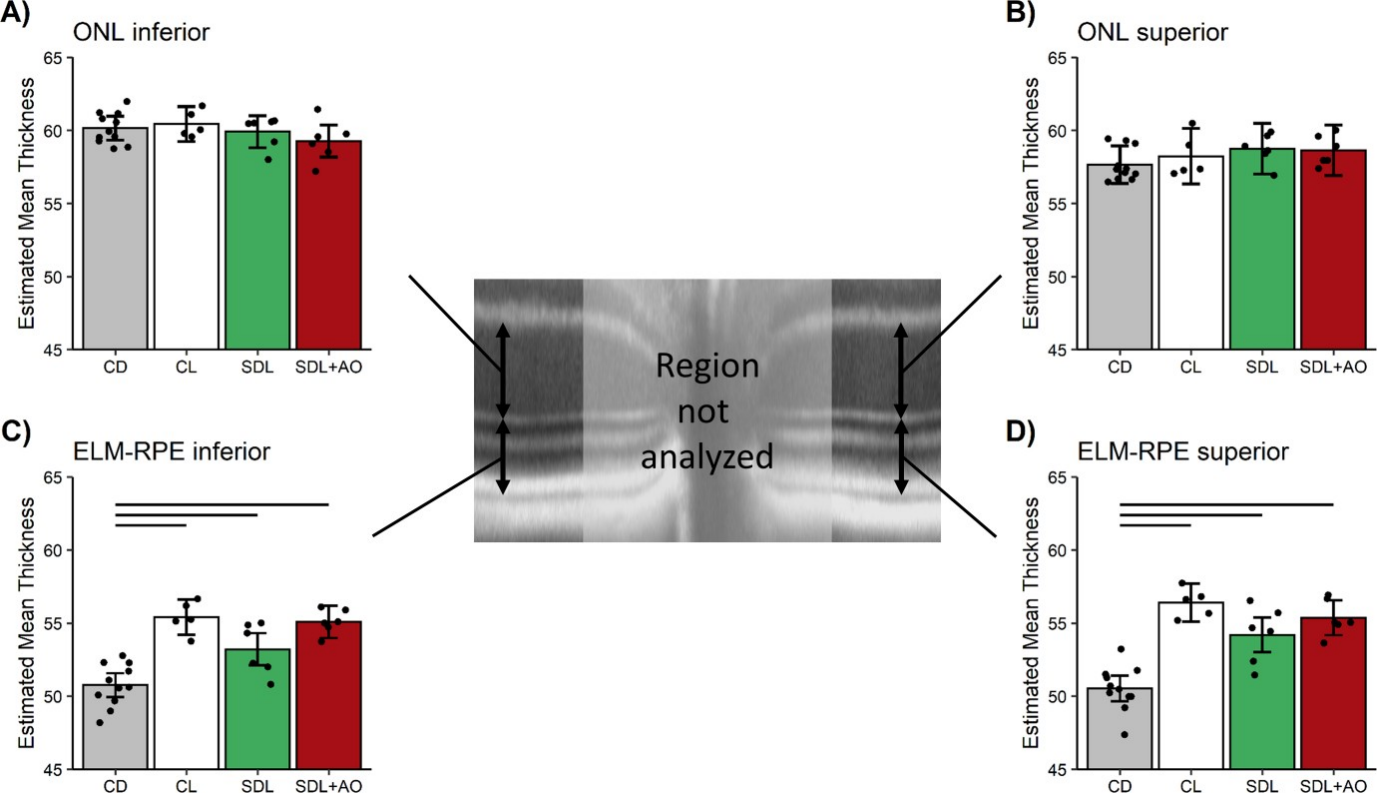
5 hr post-sildenafil in light-adaption mice (SDL, Fig 2) shows light-like ELM-RPE. Modeled A) ONL inferior retina, B) ONL superior retina, C) ELM-RPE thickness inferior retina, and D) ELM-RPE thickness superior retina in uninjected control dark (CD, n = 11 mice, grey bar), control light (CL, n = 5 mice, white bar), SDL+saline (SDL, n = 6 mice, green bar), and SDL+AO (n = 6 mice, red bar) in the two different retinal regions. ONL is unresponsive to condition. In SDL+saline and SDL+AO groups ELM-RPE thickness is not different from CL (and was thicker than CD); no evidence for oxidative stress was found. The points in each plot represent the estimated mean for each mouse based on the model. Error bars indicate 95% confidence intervals. Note the same control bars are presented in each graph to facilitate comparisons.

5 hr after sildenafil administration, the ELM-RPE thickness in the central retina (± 350–630 μm from the optic nerve head) was not different (p > 0.05) from light-adapted levels of control mice and thicker than dark-adapted levels (Fig 6 and S5 Fig in S5 File for raw data); again, anti-oxidants had no effect (p > 0.05) in the sildenafil-treated mice (Fig 6 and S5 Fig in S5 File for the raw data) [24]. Results with a field-of-view ± 350–1191 μm from the optic nerve head (S6 Fig in S5 File) are consistent with the central retina findings.

ONL thickness was invariant (p > 0.05) to sildenafil or anti-oxidants (Fig 6 for ± 350–630 μm from the optic nerve head; ± 350–1191 μm from the optic nerve head data are shown in S6 Fig in S5 File), supporting a specific sildenafil response by the ELM-RPE region.

### Testing dark-adapted mouse for outer retina oxidative stress 1 hr post-sildenafil (SDD)

#### QUEST MRI

**Fig 7:**
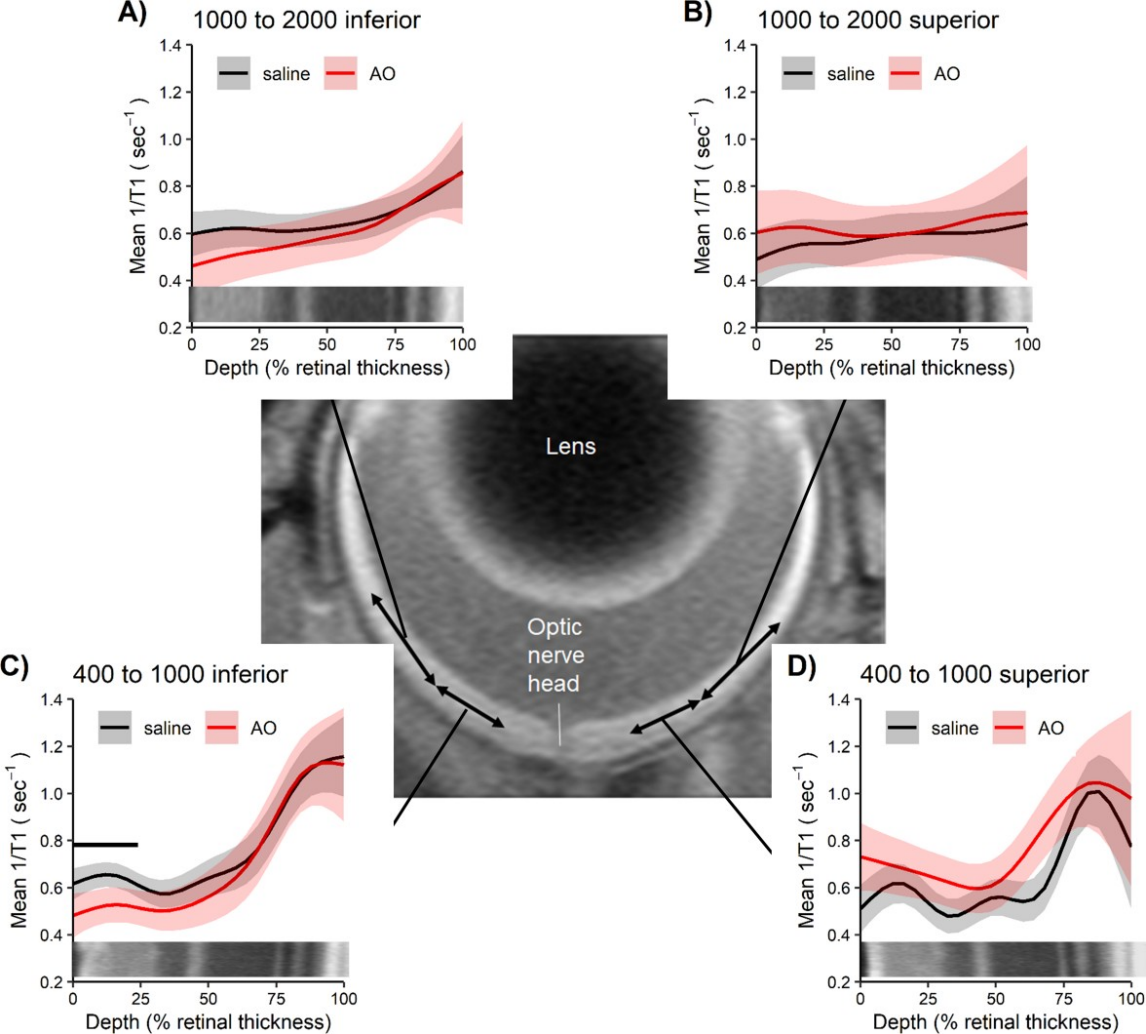
QUEST MRI in dark-adapted mice do not show outer oxidative stress. Modelled 1/T1 profiles approximately 1 hour post-sildenafil IP in dark-adapted mice given either saline (n = 6 mice, black line) or anti-oxidants (AO, n = 3 mice, red line) in these four retinal regions: A) 1000 to 2000 inferior, B) 1000 to 2000 superior, C) 400–1000 inferior, and D) 400–1000 superior; representative OCT images (bottom of each graph) provide spatial orientation. Only significant (horizontal black bar) AO reduction in 1/T1 indicative of oxidative stress are shown. Each profile has a solid line indicating the mean and a shaded region indicating 95% confidence intervals.

No evidence (p > 0.05) for oxidative stress was found in superior or inferior dark-adapted retina in any layers (Fig 7 and S7 Fig in S5 File for raw data). For completeness, we note that the inferior inner retina (0–24% depth) did show evidence (p < 0.05) for oxidative stress (i.e., Fig 7C).

#### QUEST OCT

**Fig 8:**
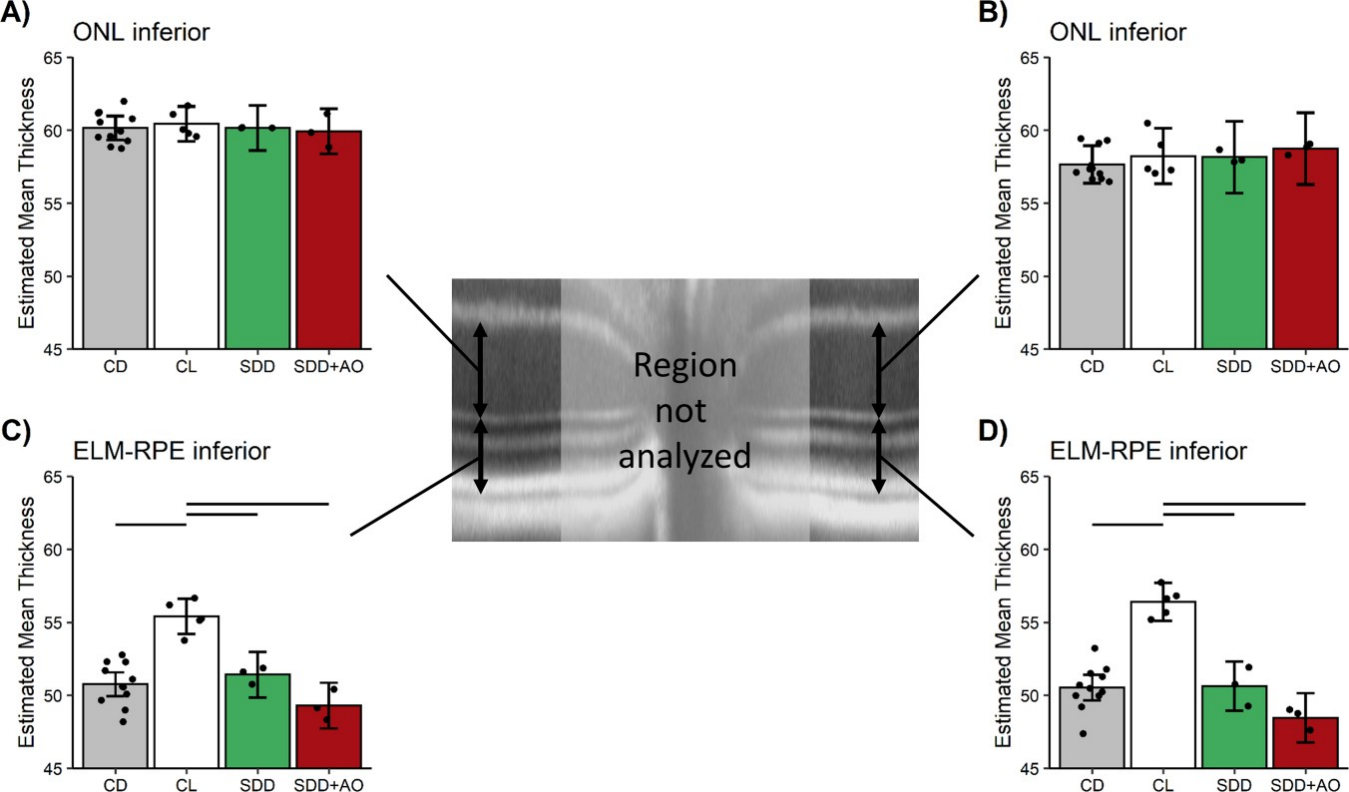
1 hr post-sildenafil in dark-adapted mice (SDD, Fig 2) shows thinner ELM-RPE. Modeled A) ONL inferior retina, B) ONL superior retina, C) ELM-RPE thickness inferior retina, and D) ELM-RPE thickness superior retina in uninjected control dark (CD, n = 11 mice, grey bar), control light (CL, n = 5 mice, white bar), SDD+saline (SDD, n = 3 mice, green bar), and SDD+AO (n = 3 mice, red bar) in the two different retinal regions. ONL is constant regardless of condition. In SDD+saline and SDD+AO groups, ELM-RPE thickness is not different from CD (and was thinner than CL); no evidence for oxidative stress was found. The points in each plot represent the estimated mean for each mouse based on the model. Error bars indicate 95% confidence intervals. Note the same control bars are presented in each graph to facilitate comparisons.

In the dark, the ELM-RPE thickness in the central retina (± 350–630 μm from the optic nerve head) was not different (p > 0.05) from dark-adapted levels of control mice and was thinner, as expected, than light-adapted levels independent of administration of anti-oxidants (Fig 8 and S8 Fig in S5 File for raw data) [24]. Comparable outcomes were observed with a field-of-view ± 350–1191 μm from the optic nerve head (S9 Fig in S5 File).

In addition, the ONL thickness was constant regardless of treatment with sildenafil or anti-oxidants (Fig 8 for ± 350–630 μm from the optic nerve head; ± 350–1191 μm from the optic nerve head shown in S9 Fig in S5 File).

### Testing visual performance 1, 5, and 24 hr post-sildenafil

#### QUEST OKT

**Fig 9:**
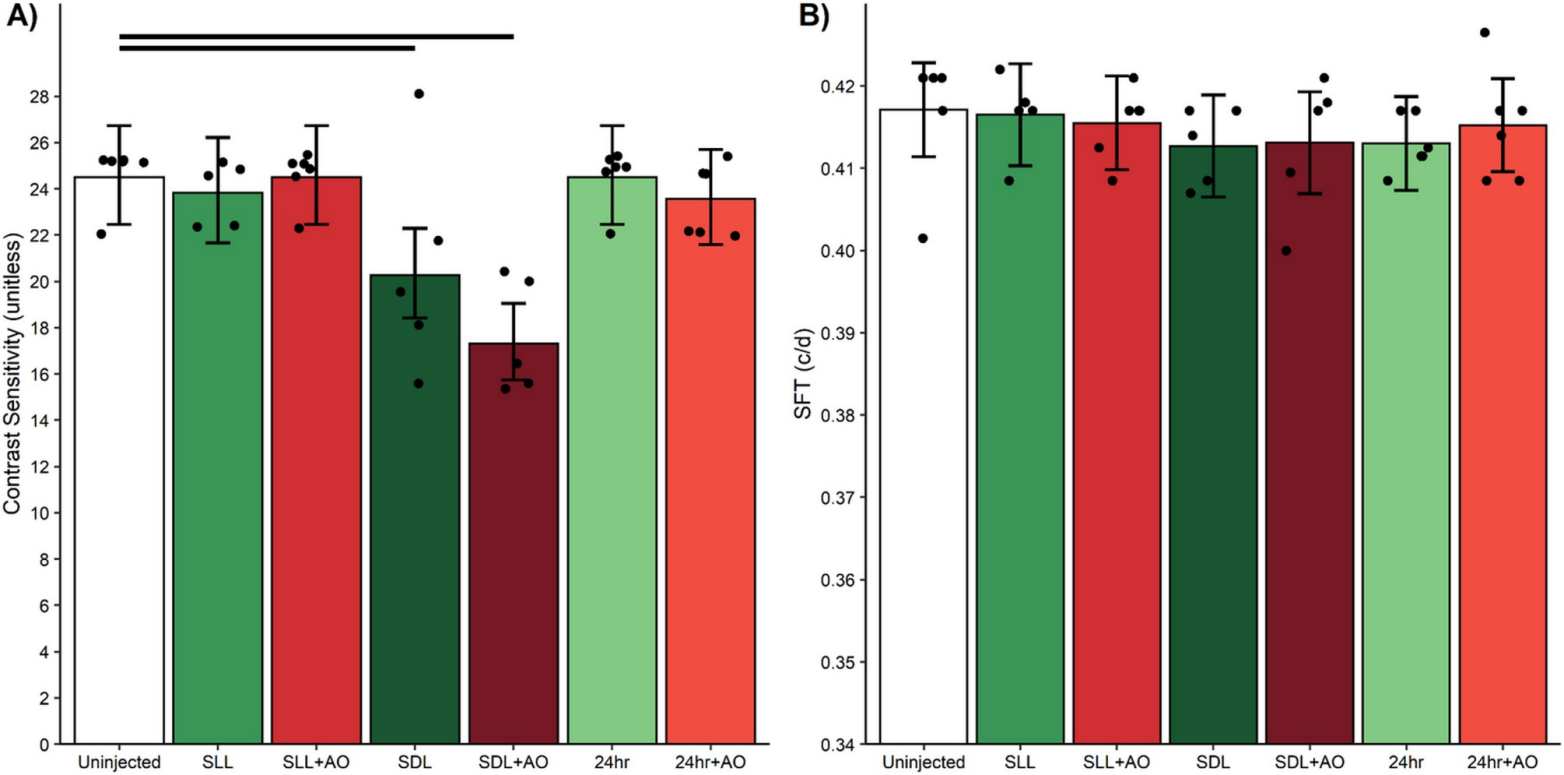
Contrast sensitivity is transiently reduced at 5 hr post-sildenafil. Compared to uninjected 5 hr light adapted mice (white bar), sildenafil + saline (green bars) A) decreased contrast sensitivity only under SDL conditions but B) did not change spatial frequency threshold (SFT). AO (red bars) did not correct this reduced contrast sensitivity. Black horizontal bars indicate significant differences (P < 0.05). The points in each plot represent the estimated mean for each mouse based on the model. Error bars are 95% confidence intervals. Number of mice used per group: uninjected, n = 6 mice; SLL+saline, n = 5 mice; SLL+AO, n = 6 mice; SDL+saline, n = 5 mice; SDL+AO, n = 5 mice; 24 hour+saline, n = 6 mice; 24 hour+AO, n = 6 mice.

At 5 hr post-sildenafil treatment, CS was lower-than-normal (p < 0.05) and not corrected by anti-oxidants (Fig 9). No change (p > 0.05) in CS from control values was noted at 1 hr or 24 post-sildenafil. SFT was unaffected by both sildenafil ± anti-oxidants in all groups (Fig 9).

## Discussion

In this study, we find evidence for a spatially limited oxidative stress response to sildenafil, and that sildenafil-impaired vision cannot be rescued by AO. Therefore, it is likely that sildenafil-induced vision impairment is caused by mechanisms other than oxidative stress, a topic for future study.

### Sildenafil and retina oxidative stress

In this study, we found no evidence to support the first part of our hypothesis that systemic sildenafil can cause widespread outer retinal oxidative stress in light-adapted mice (Figs 3 and 4). Unexpectedly, the oxidative stress (as measured by QUEST MRI *in vivo* and DCF *ex vivo*) at 1 hr post-treatment was found most prominently in superior peripheral outer retina (Figs 3 and 4). Intriguingly, we note that other retinal regions (e.g., superior central outer retina) show different oxidative stress results by QUEST MRI and DCF. These different spatial outcomes do not appear to be due to different detection sensitivities because, for example, a third method, QUEST OCT, did not show evidence for oxidative stress in inferior or superior central retina (Fig 5). Alternatively, we note that QUEST MRI detects paramagnetic free radicals and is unable to detect non-paramagnetic species like H_2_O_2._ However, DCF detects both free radicals and H_2_O_2_ [45]. We also note that QUEST OCT detects high levels of superoxide but its sensitivity to other reactive oxygen species is unknown [20, 24, 44]. More work is needed to unravel which reactive oxygen species contribute to the positive QUEST MRI, DCF, and QUEST OCT results.

Regionally specific oxidative stress has been reported in PDE 6 mutant rd10 mice and in other models [31, 33, 55–57]. We note that non-invasive measures of oxidative stress measured by QUEST MRI, QUEST OCT, and QUEST OKT are only revealed with AO injection, and the use of saline controls is thus better than un-injected controls. The present results are consistent with the notion that seemingly homogeneous population of neurons (in this case rod photoreceptors) can have substantial within-class heterogeneity [55]. Limitations of the present study include not considering sex and age, two important biological variables; more work is now needed in female and older mice.

**Fig 10:**
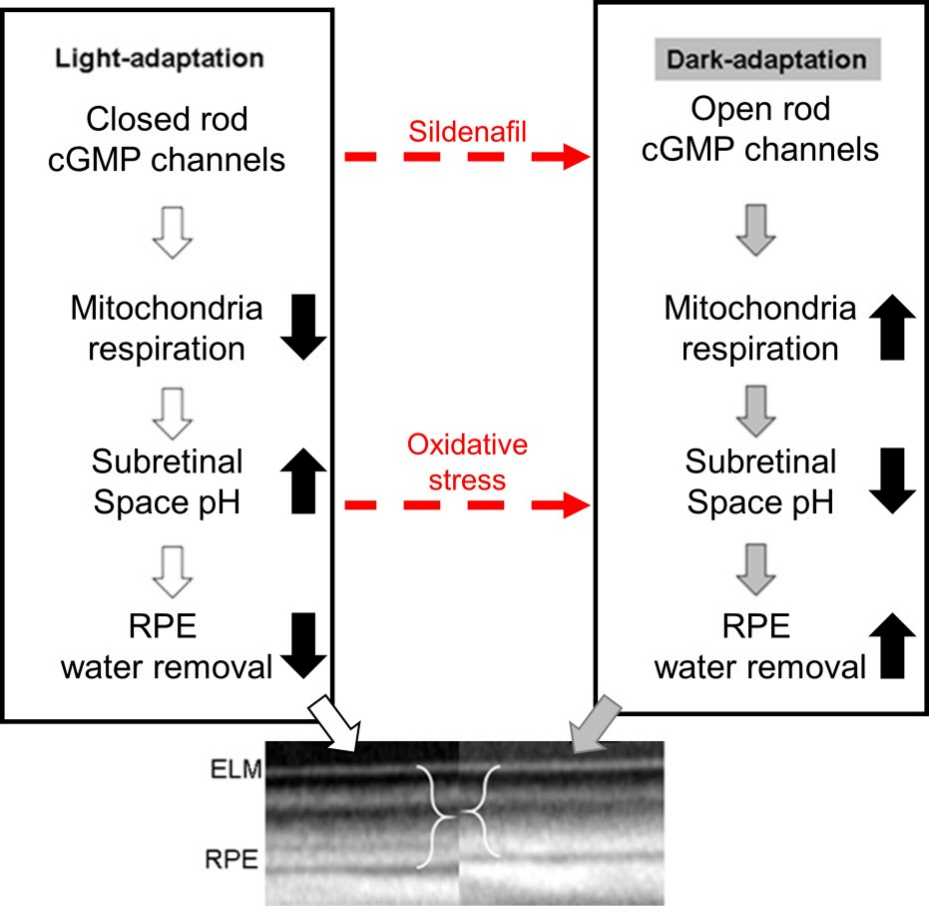
Working model of factors that regulate the ELM-RPE thickness. Oxidative stress is hypothesized to induce an acidosis (dotted red line) that would be expected to convert a thicker “light” ELM-RPE phenotype into a thinner “dark-like” phenotype (see text for details); this expectation is supported experimentally [24, 44, 63].

As noted above, QUEST OCT found no evidence for oxidative stress in the ELM-PRE region of the central retina [24, 29, 33]. QUEST OCT relies on the fact that oxidative stress causes a thinner, dark-like ELM-RPE thickness even in the light, perhaps due to induced acidosis [58–60]. This phenotype comes about because the ELM-RPE thickness is modulated by a signaling pathway in which rod photoreceptor cells consume more energy in the dark than in the light [26, 27, 32, 44, 61, 62]. As a result, there is a greater production of waste-water and CO_2_ that acidifies the subretinal space [26, 27, 32, 44, 61, 62]. In turn, this causes an upregulation of pH-sensitive water-removal co-transporters on the apical portion of the RPE (Fig 10) [26, 27, 32, 44, 61, 62]. Thus, oxidative stress is indicated if in light-adapted mice, anti-oxidants convert a dark-like thin subretinal space into a thicker light-like level (Fig 10), an outcome not found in this study (Figs 5, 6, 8) [24].

Mechanistically, the evoked oxidative stress at 1 hr post-treatment (at the presumptive peak of plasma sildenafil and associated cGMP content) is hypothesized to occur via focal inhibition of photoreceptor PDE 6 activity [12]. In this scenario (summarized in Fig 10), elevating cGMP levels in light-adapted mice causes sustained opening of rod photoreceptor outer segment cyclic nucleotide-gated channels, an energy-intensive event [64]. As a result of this increase in mitochondrial respiration, the subretinal space fills with acidified waste-water [14, 17–19, 24, 28, 65]. The lowering of the subretinal space pH upregulates water removal co-transporters in RPE with the end result of dehydrating / thinning of the ELM-RPE space (Fig 10) [14, 17–19, 24, 28, 65]. Evidence for subretinal space thinning following sildenafil is shown in Fig 3 [14, 17–19, 24, 28, 65]. The associated increase in mitochondrial activity can cause production of free radicals in excess of anti-oxidant defenses [16]. The above may also provide a possible explanation for the oxidative stress measured in the inferior inner central retina (a PDE 5-rich region) of dark-adapted mice (Figs 4 and 6) [7, 12, 66]. While sustained cGMP content can cause photoreceptor cell death, the induced focal oxidative stress in this study (Fig 3) appears to be non-toxic since visual performance was normal 24 hours after the sildenafil treatment [16–19, 31, 65, 67]. Since the outer retina is avascular, the focal oxidative stress in posterior retina is unrelated to a sildenafil effect on endothelial cells [68]. There are reports, however, in non-retinal disease models that chronic treatment with sildenafil shows anti-oxidant effects [69, 70]. More work is needed to understand the events leading to the localized oxidative stress *in vivo* measured in the present investigation.

### Sildenafil and visual performance

We further found novel evidence that sildenafil can temporarily impair contrast sensitivity but not spatial frequency thresholds (i.e., acuity) in healthy C57BL/6 mice (Fig 9). This visual performance decline in response to sildenafil was not corrected with anti-oxidants, suggesting a non-oxidative stress etiology. The transient nature of the response (Fig 9) may help understand why sildenafil’s effect on retinal electrophysiologic indices from experimental models has been contradictory [6–9, 12, 13].

Intriguingly, the induced visual performance declines also did not seem linked with higher rod cGMP levels. In particular, sildenafil’s plasma concentration peaks at about 1 hr post-injection followed by clearance with a half-life of ~4 hr; effects on retinal electrophysiology are reported to be gone by 24 hr post-injection [6, 12, 66, 71]. Sildenafil’s content in the retina is ~2-fold higher than in the plasma and so the resulting higher retinal cGMP levels are expected to follow a similar time course as sildenafil in the plasma [9]. Indeed, in this study, a smaller rod ELM-RPE thickness was evident at 1 hr post-sildenafil with recovery to thicker light-like levels by 5 hr (Fig 5) in-line with the expected changes in cGMP levels (Fig 10) [6, 9, 12, 66, 71]. However, contrast sensitivity was normal at 1 hr post-sildenafil (i.e., at the presumptive maximum plasma and retinal levels of sildenafil and cGMP, respectively) (Fig 9) [12]. On the other hand, visual performance was lowest at 5 hr post-treatment (i.e., when levels are roughly at half-maximum) (Fig 9) [12]. The present data are insufficient to explain the temporal mismatch between lower contrast sensitivity and rod cGMP levels, especially since cones are more sensitive to sildenafil inhibition than rods [12]. Additional studies are needed to investigate whether, for example, this mismatch has contributions from retinal sildenafil P450 metabolism or from PDE inhibition in other parts of the brain [10, 72–74].

### Summary

Sildenafil induced spatially localized photoreceptor oxidative stress based on i) the QUEST MRI and DCF data showing prominent signal from superior peripheral outer retina dominated by rods in the light demonstrating oxidative stress [75], ii) the lack of evidence for oxidative stress in central outer retina as measured by QUEST OCT, and iii) lack of anti-oxidant response on OKT cone-based visual performance testing. Thus, we find no evidence for sildenafil-induced oxidative stress in rods panretinally nor was oxidative stress related to sildenafil-induced impairment of visual performance in wildtype mice. In addition, the present findings do not support a role for loss-of-function in PDE 6 *per se* as causative for oxidative stress in dark-reared rd10 mice stress [31]. A novel observation from this study is that ELM-RPE thickness appears to be an indicator of cGMP level changes at least following sildenafil administration; conventional cGMP assays reflect whole retina levels rather than that in the ELM-RPE [76]. This observation might thus address the need for novel cGMP imaging biomarkers in, for example, hereditary photoreceptor degeneration disease, age-related macular degeneration, and neurodegenerative diseases, such as Alzheimer’s disease [2, 4, 77].

## Supporting information

S1 File Supplement File. The supplement file contains additional details about the statistical methods used, supplemental tables, supplemental figures, and code for all analyses.
(ZIP)

S2 File Data for Figs 3 and 7. This file contains the summary data and code needed to generate Figs 3 and 7.
(ZIP)

S3 File Data for Figs 5, 6, and 8. This file contains the summary data and code needed to generate Figs 5, 6 and 8.
(ZIP)

S4 File Data for Fig 9. This file contains the summary data and code needed to generate Fig 9.
(ZIP)

S5 File (DOCX)
