# Supplementary material for: Sildenafil-evoked photoreceptor oxidative stress in vivo is unrelated to impaired visual performance in mice
Source: PLoS One. 2021 Mar 4;16(3):e0245161. doi: 10.1371/journal.pone.0245161 (PMC7932139; doi:10.1371/journal.pone.0245161)

**Sildenafil-evoked photoreceptor oxidative stress *in vivo* is**

**unrelated to impaired visual performance in mice**

**Supplementary Material**

Bruce A. Berkowitz^1*^, Robert H. Podolsky^2^, Karen Lins Childers^2^, Robin Roberts^1^, Hailey Olds^1a^, Joydip Joy^1a^, Collin Richards^1a^, Tilman Rosales^1^, Michael Schneider^1^, Brennan Schilling^1b^, Arthur Orchanian^1b^, Emma Graffice^1^, Kenan Sinan^1^, Haohua Qian^3^, and Lamis Harp^1^

^1^Department of Ophthalmology, Visual and Anatomical Sciences, Wayne State University School of Medicine, Detroit, MI 48201; ^2^Beaumont Research Institute, Beaumont Health, Royal Oak, MI 48073; ^3^Visual Function Core National Eye Institute, National Institutes of Health, Bethesda, Maryland

**Supplementary Material Contents:**

Contents

[Supplementary Methods: 4](#_Toc50643551)

[Supplementary Tables: 9](#_Toc50643552)

[Table S1. Fixed effect parameter estimates for the model fit to the 1/T1. 9](#_Toc50643553)

[Table S2. Covariance parameters estimated for the model fit to 1/T1. 12](#_Toc50643554)

[Table S3. Fixed effect parameter estimates for the model fit to ONL thickness on the inferior side. 13](#_Toc50643555)

[Table S4. Covariance parameters estimated for ONL thickness on the inferior side. 14](#_Toc50643556)

[Table S5. Fixed effect parameter estimates for the model fit to ONL thickness on the superior side. 15](#_Toc50643557)

[Table S6. Covariance parameters estimated for ONL thickness on the superior side. 17](#_Toc50643558)

[Table S7. Fixed effect parameter estimates for the model fit to ELM-RPE thickness on the inferior side. 17](#_Toc50643559)

[Table S8. Covariance parameters estimated for ELM-RPE thickness on the inferior side. 19](#_Toc50643560)

[Table S9. Fixed effect parameter estimates for the model fit to ELM-RPE thickness on the superior side. 19](#_Toc50643561)

[Table S10. Covariance parameters estimated for ELM-RPE thickness on the superior side. 21](#_Toc50643562)

[Table S11. Fixed effect parameter estimates for the model fit to superoxide levels. 21](#_Toc50643563)

[Table S12. Covariance parameters estimated for the model fit to superoxide levels. 21](#_Toc50643564)

[Table S13. Fixed effect parameter estimates for the model fit to contrast sensitivity. 22](#_Toc50643565)

[Table S14. Covariance parameters estimated for the model fit to contrast sensitivity. 22](#_Toc50643566)

[Table S15. Fixed effect parameter estimates for the model fit to spatial frequency threshold (SFT). 22](#_Toc50643567)

[Table S16. Covariance parameters estimated for the model fit to spatial frequency threshold (SFT). 22](#_Toc50643568)

[Supplementary Figures: 23](#_Toc50643569)

[Figure S1. QUEST MRI showing oxidative stress localized to peripheral superior retina 23](file:///C:\Users\bh188799.BH\Documents\home\papers\Berkowitz\Sildenafil_0520\Berkowitz%20Sildenafil%20PLoS%20One%20Supplement.docx#_Toc50643570)

[Figure S2. Superoxide levels do not change with sildenafil treatment 24](file:///C:\Users\bh188799.BH\Documents\home\papers\Berkowitz\Sildenafil_0520\Berkowitz%20Sildenafil%20PLoS%20One%20Supplement.docx#_Toc50643571)

[Figure S3: 1 hr post sildenafil after 4 hr of light-adaption mice (SLL, Figure 2) shows thinner dark-like ELM-RPE. 25](file:///C:\Users\bh188799.BH\Documents\home\papers\Berkowitz\Sildenafil_0520\Berkowitz%20Sildenafil%20PLoS%20One%20Supplement.docx#_Toc50643572)

[Figure S4: 1 hr post sildenafil after 4 hr of light-adaption mice (SLL, Figure 2) shows thinner, dark-like ELM-RPE in extended field of view 26](file:///C:\Users\bh188799.BH\Documents\home\papers\Berkowitz\Sildenafil_0520\Berkowitz%20Sildenafil%20PLoS%20One%20Supplement.docx#_Toc50643573)

[Figure S5: 5 hr post sildenafil in light-adapted mice (SDL) shows light-like ELM-RPE. 27](file:///C:\Users\bh188799.BH\Documents\home\papers\Berkowitz\Sildenafil_0520\Berkowitz%20Sildenafil%20PLoS%20One%20Supplement.docx#_Toc50643574)

[Figure S6: 5 hr post sildenafil in light-adaption mice (SDL, Figure 2) shows light-like ELM-RPE in extended field of view 28](file:///C:\Users\bh188799.BH\Documents\home\papers\Berkowitz\Sildenafil_0520\Berkowitz%20Sildenafil%20PLoS%20One%20Supplement.docx#_Toc50643575)

[Figure S7. QUEST MRI in dark-adapted mice do not show outer oxidative stress. 29](file:///C:\Users\bh188799.BH\Documents\home\papers\Berkowitz\Sildenafil_0520\Berkowitz%20Sildenafil%20PLoS%20One%20Supplement.docx#_Toc50643576)

[Figure S8: 1 hr post sildenafil in dark-adapted mice shows thinner ELM-RPE. 30](file:///C:\Users\bh188799.BH\Documents\home\papers\Berkowitz\Sildenafil_0520\Berkowitz%20Sildenafil%20PLoS%20One%20Supplement.docx#_Toc50643577)

[Figure S9: 1 hr post sildenafil in dark-adapted mice (SDD, Figure 2) shows thinner ELM-RPE in extended field of view. 31](file:///C:\Users\bh188799.BH\Documents\home\papers\Berkowitz\Sildenafil_0520\Berkowitz%20Sildenafil%20PLoS%20One%20Supplement.docx#_Toc50643578)

# **Supplementary Methods**:

Data are presented as mean ± 95% confidence interval, and a significance level of 0.05 was used for all analyses. We used mixed linear models to analyze all outcomes (1/T1, superoxide, OCT layer thickness, and OKT) since all outcomes had repeated measures for each mouse. We used Proc Mixed and Proc Glimmix of SAS 9.4 (SAS software, Cary, NC, USA) to fit all models, and we used the Kenward-Roger method to calculate degrees of freedom. We used the same modeling strategy for both MRI profile data (1/T1) and OCT layer thickness, using random coefficients and restricted cubic splines to model and compare mouse-specific profiles between groups. The general model fit was of the form,

$$y_{ij}=\left( \beta_{0}+u_{0i} \right)+\sum_{m=1}^{M} \left( \beta_{m}+u_{mi} \right)x_{ijm}+\sum_{k=0}^{K} \left( \beta_{spline_{k}}+u_{spline_{k}i} \right)x_{ijs_{k}}+\sum_{m=1}^{M} \sum_{k=1}^{K} \left( \beta_{spline_{k}m}+u_{spline_{k}m,i} \right){x_{ijm}x}_{ijs_{k}}+\epsilon_{ij},$$

where $y_{ij}$ is the $j$th observation for the $i$th mouse, there are $M$ coefficients for fixed effects, $K$ internal knot locations, $x_{ijs_{0}}=$ depth/distance $\boldsymbol{u}_{i}=\left[ \begin{matrix} u_{0i} & \boldsymbol{u}_{\boldsymbol{Mi}} & \boldsymbol{u}_{spline_{K}i} & \boldsymbol{u}_{spline_{K}M,i} \end{matrix} \right]^{T}$, and each of $\boldsymbol{u}_{\boldsymbol{Mi}}$**,** $\boldsymbol{u}_{spline_{K}i}$, and $\boldsymbol{u}_{spline_{K}M,i}$ being vectors of all random coefficients of a given type. We assume that $\boldsymbol{u}_{i}\sim N(\boldsymbol{0},\boldsymbol{\Sigma}$), with $\boldsymbol{\Sigma}$ being a general covariance matrix. Further, we assume that $\epsilon_{ij}\sim N\left( 0,\sigma_{e}^{2} \right)$, with all observations being independent conditional on the random effects. This approach allows for associations in random coefficients, as could result when a larger coefficient for some knot locations would need to be offset by smaller coefficients at other locations.

The model above shows the general form, and we never attempted to fit a model having random coefficients for all effects. The actual random coefficients and the number of knots included in any model was determined by our modeling approach. We began our modeling by selecting the number of knots using only random effects needed based on the design of the study. For example, a random coefficient for side would be included when both inferior and superior sides were being modeled, but no random spline coefficients would be included. The number of knots was first selected by fitting separate models for each group of mice separately. The Akaike and Schwarz Bayesian information criteria (AIC and BIC) were used to identify the model with the fewest knots by combining the AIC and BIC across all groups, with the overall AIC being the sum of the AIC for all models, and the overall BIC being calculated as $BIC=\sum_{g} -2L_{g}+G*\left( K*M+R \right)+log(n)$, where $L_{g}$ is the log-likelihood of the $g$th group, $G$ is the number of groups, $K$ is the number of knots, $M$ is the number of fixed parameters in the model, and $R$ is the number of random parameters in the model.

We next selected additional random coefficients for side (indicator variable for superior side), region (indicator of 1000-2000 μm region), and location-specific coefficients (cubic spline coefficients) based on AIC and BIC in a full model. The resulting selected model with was then used to test for the fixed effects for each model, and non-significant interactions were removed to obtain the final model. The final model was then used to estimate mean profiles for all experimental conditions, and location-specific mean differences based on appropriate contrasts.

For 1/T1, each mouse was measured at 26 depths on two sides in two regions, resulting in 104 observations per mouse. We used Proc Mixed to fit the model for 1/T1 which included seven knots and the fixed effects for anti-oxidant (saline vs MB/ALA), side (inferior vs superior), condition (dark vs light), region (400-1000 μm vs 1000-2000 μm), as well as all interactions among these fixed effects and the locations. The final model included the random coefficients for side; region; linear relationship with depth; the fourth knot coefficient; all two-way interactions among side, region, and the fourth knot coefficient; and the interaction between region and the linear relationship with depth. Thus, the final model can be written as

$$y_{ij}=\left( \beta_{0}+u_{0i} \right)+{(\beta}_{1}+u_{1i}) side_{ij}+\beta_{2}AO_{ij}+\left( \beta_{3}+u_{3i} \right) region_{ij}+\beta_{4}condition_{ij}+\beta_{5}side_{ij}AO_{ij}+\beta_{6}side_{ij}region_{ij}+\beta_{7}side_{ij}{condition}_{ij}+\beta_{8}AO_{ij}region_{ij}+\beta_{9}AO_{ij}condition_{ij}+\beta_{10}region_{ij}condition_{ij}+\beta_{11}side_{ij}AO_{ij}region_{ij}+\beta_{12}side_{ij}AO_{ij}{condition}_{ij}+\beta_{13}side_{ij}region_{ij}condition_{ij}+\beta_{14}AO_{ij}region_{ij}condition_{ij}+\sum_{k=0}^{5} \left( \beta_{spline_{k}} \right)x_{ijs_{k}}+u_{spline_{0}i}x_{ijs_{0}}+u_{spline_{4}i}x_{ijs_{4}}+\sum_{k=0}^{5} \left( \beta_{spline_{k}1} \right)side_{ij}x_{ijs_{k}}+\sum_{k=0}^{5} \left( \beta_{spline_{k}2} \right){AO}_{ij}x_{ijs_{k}}+\sum_{k=0}^{5} \left( \beta_{spline_{k}3} \right){region}_{ij}x_{ijs_{k}}+\sum_{k=0}^{5} \left( \beta_{spline_{k}4} \right){condition}_{ij}x_{ijs_{k}}+\sum_{k=0}^{5} \left( \beta_{spline_{k}12} \right)side_{ij}AO_{ij}x_{ijs_{k}}+\sum_{k=0}^{5} \left( \beta_{spline_{k}13} \right)side_{ij}{region}_{ij}x_{ijs_{k}}+\sum_{k=0}^{5} \left( \beta_{spline_{k}14} \right)side_{ij}{condition}_{ij}x_{ijs_{k}}+\sum_{k=0}^{5} \left( \beta_{spline_{k}23} \right){region}_{ij}AO_{ij}x_{ijs_{k}}+\sum_{k=0}^{5} \left( \beta_{spline_{k}24} \right){region}_{ij}{condition}_{ij}x_{ijs_{k}}+\sum_{k=0}^{5} \left( \beta_{spline_{k}34} \right)AO_{ij}condition_{ij}x_{ijs_{k}}+\sum_{k=0}^{5} \left( \beta_{spline_{k}123} \right)side_{ij}AO_{ij}region_{ij}x_{ijs_{k}}+\sum_{k=0}^{5} \left( \beta_{spline_{k}124} \right)side_{ij}AO_{ij}{condition}_{ij}x_{ijs_{k}}+\sum_{k=0}^{5} \left( \beta_{spline_{k}134} \right)side_{ij}{region}_{ij}{condition}_{ij}x_{ijs_{k}}+\sum_{k=0}^{5} \left( \beta_{spline_{k}234} \right){AO}_{ij}{region}_{ij}{condition}_{ij}x_{ijs_{k}}+u_{spline_{0}region,i}{region}_{ij}x_{ijs_{0}}+u_{spline_{4}side,i}{side}_{ij}x_{ijs_{4}}+u_{spline_{4}region,i}{region}_{ij}x_{ijs_{4}}+\epsilon_{ij},$$

The parameter estimates for the model are shown in Table S1 and Table S2. We used contrasts of the parameter estimates to test whether the MB/ALA effect was statistically significant at each measured depth (see “estimate” statements in SAS code).

For the OCT data, each mouse was measured at 720 distances from the ONH. We fit four models to the OCT data using Proc Glimmix, a separate model for each outcome (changes in ELM-RPE or ONL) and for inferior and superior sides. All models included fixed effects for group (light-adapted control, CL; dark-adapted control, CD; SLL; SLL+MB/ALA; SDL; SDL+MB/ALA; SDD, SDD+MB/ALA), values for the spline coefficients for the distance from the ONH and the interaction between distance from the ONH and group. While the number of knots varied across the four models, all four models were of the form

$$y_{ij}=\left( \beta_{0}+u_{0i} \right)+\sum_{g=1}^{7} \beta_{g}x_{ijg}+\sum_{k=0}^{K} \left( \beta_{spline_{k}}+u_{spline_{k}i} \right)x_{ijs_{k}}+\sum_{m=1}^{M} \sum_{g=1}^{7} \beta_{spline_{k}g}{x_{ijg}x}_{ijs_{k}}+\epsilon_{ij},$$

where there are seven indicator variables for group ($x_{ijg}$) and the number of knots is specific to the outcome and side. Random coefficients were included for at most two of the knot locations. The number of knots and random coefficients used for the four final models are shown in Table 1. The parameter estimates for the four models are shown in Tables S3-S10 below. Contrasts were used with each final model to calculate a mean integrated across the entire layer. These contrasts were also used to compare the integrated means among the groups. We compared each group to the both controls and MB/ALA was compared only within a group (e.g., SLL vs SLL+MB/ALA). We used the Holm procedure to adjust for multiple comparisons for each focal group (SLL, SDL, and SDD).

Superoxide levels were measured three times each in three to four batches resulting in each mouse having 9-12 measurements. The measurements were done over two days, with three mice being treated with saline and two mice being treated with sildenafil on the first day, and with two mice being treated with saline and three mice being treated with sildenafil on the second day. As such, superoxide levels were analyzed using a linear mixed model that included the fixed effect of treatment (saline, sildenafil) and random intercepts for mouse nested within treatment, batch nested within mouse and treatment, and day. The parameter estimates are shown in Tables S11 and S12 in the supplement. The primary test for this analysis was of the treatment effect (saline vs. sildenafil).

OKT was measured once per side, resulting in only two observations per mouse. As such, we used generalized linear mixed models to analyze both OKT measurements. For both acuity and contrast sensitivity, we included the fixed effect of group (saline, SLL, SLL+MB/ALA, SDL, SDL+MB/ALA, 24hr, 24hr+MB/ALA. Only a random intercept for mouse nested within group was included for these models. We used a normal distribution with the identity link for acuity and a gamma distribution with the log link for contrast sensitivity. The parameter estimates for the fit models are shown in Tables S13-S16 in the supplement. As with OCT distances, we compared each experimental group to saline, and compared the MB/ALA treatment within a focal group. We used the Holm procedure to adjust for multiple comparisons.

# **Supplementary Tables:**

## Table S1. Fixed effect parameter estimates for the model fit to the 1/T1.

| Effect | treat | side | condition | region | Estimate | Standard Error | DF | t Value | Pr > \|t\| |
| --- | --- | --- | --- | --- | --- | --- | --- | --- | --- |
| Intercept |  |  |  |  | 0.4423 | 0.2012 | 757 | 2.2 | 0.0283 |
| treat | AO |  |  |  | 0.4023 | 0.2725 | 757 | 1.48 | 0.1403 |
| side |  | L |  |  | 0.3176 | 0.2548 | 596 | 1.25 | 0.2131 |
| treat*side | AO | L |  |  | -0.3842 | 0.3385 | 596 | -1.14 | 0.2568 |
| condition |  |  | dark |  | 0.6555 | 0.3087 | 757 | 2.12 | 0.034 |
| treat*condition | AO |  | dark |  | -0.9226 | 0.4546 | 757 | -2.03 | 0.0428 |
| side*condition |  | L | dark |  | -0.5633 | 0.371 | 596 | -1.52 | 0.1295 |
| treat*side*condition | AO | L | dark |  | 0.7514 | 0.5221 | 596 | 1.44 | 0.1506 |
| region |  |  |  | 1000to2000 | 0.2715 | 0.2475 | 1538 | 1.1 | 0.273 |
| treat*region | AO |  |  | 1000to2000 | -0.3144 | 0.3451 | 1538 | -0.91 | 0.3624 |
| side*region |  | L |  | 1000to2000 | -0.2483 | 0.3387 | 1506 | -0.73 | 0.4637 |
| treat*side*region | AO | L |  | 1000to2000 | 0.1781 | 0.4543 | 1506 | 0.39 | 0.6951 |
| condition*region |  |  | dark | 1000to2000 | -0.6327 | 0.3824 | 1538 | -1.65 | 0.0982 |
| treat*conditi*region | AO |  | dark | 1000to2000 | 0.8262 | 0.5738 | 1538 | 1.44 | 0.1501 |
| side*conditio*region |  | L | dark | 1000to2000 | 0.4691 | 0.492 | 1506 | 0.95 | 0.3404 |
| trea*side*cond*regio | AO | L | dark | 1000to2000 | -0.6245 | 0.665 | 1506 | -0.94 | 0.3478 |
| depthc |  |  |  |  | -0.00164 | 0.004577 | 703 | -0.36 | 0.7197 |
| depthc*treat | AO |  |  |  | 0.004156 | 0.0062 | 703 | 0.67 | 0.5029 |
| depthc*side |  | L |  |  | 0.006781 | 0.005582 | 1634 | 1.21 | 0.2246 |
| depthc*treat*side | AO | L |  |  | -0.00507 | 0.007416 | 1634 | -0.68 | 0.4946 |
| depthc*condition |  |  | dark |  | 0.01337 | 0.006971 | 703 | 1.92 | 0.0555 |
| depthc*treat*conditi | AO |  | dark |  | -0.01896 | 0.01029 | 703 | -1.84 | 0.0658 |
| depthc*side*conditio |  | L | dark |  | -0.01379 | 0.00815 | 1634 | -1.69 | 0.0908 |
| dept*trea*side*condi | AO | L | dark |  | 0.01949 | 0.01146 | 1634 | 1.7 | 0.0891 |
| depthc*region |  |  |  | 1000to2000 | 0.005 | 0.005685 | 1245 | 0.88 | 0.3793 |
| depthc*treat*region | AO |  |  | 1000to2000 | -0.00442 | 0.007911 | 1245 | -0.56 | 0.5761 |
| depthc*side*region |  | L |  | 1000to2000 | -0.00425 | 0.007643 | 1634 | -0.56 | 0.5786 |
| dept*trea*side*regio | AO | L |  | 1000to2000 | 0.003542 | 0.01025 | 1634 | 0.35 | 0.7297 |
| depthc*condit*region |  |  | dark | 1000to2000 | -0.01181 | 0.008723 | 1245 | -1.35 | 0.1759 |
| dept*trea*cond*regio | AO |  | dark | 1000to2000 | 0.01679 | 0.01307 | 1245 | 1.29 | 0.199 |
| dept*side*cond*regio |  | L | dark | 1000to2000 | 0.008642 | 0.01109 | 1634 | 0.78 | 0.4358 |
| dep*tre*sid*con*regi | AO | L | dark | 1000to2000 | -0.01459 | 0.015 | 1634 | -0.97 | 0.3307 |
| depth1 |  |  |  |  | 0.07272 | 0.06232 | 1634 | 1.17 | 0.2434 |
| depth1*treat | AO |  |  |  | -0.1425 | 0.08376 | 1634 | -1.7 | 0.089 |
| depth1*side |  | L |  |  | -0.1558 | 0.07705 | 1634 | -2.02 | 0.0433 |
| depth1*treat*side | AO | L |  |  | 0.1749 | 0.1016 | 1634 | 1.72 | 0.0853 |
| depth1*condition |  |  | dark |  | -0.2735 | 0.09735 | 1634 | -2.81 | 0.005 |
| depth1*treat*conditi | AO |  | dark |  | 0.341 | 0.1434 | 1634 | 2.38 | 0.0175 |
| depth1*side*conditio |  | L | dark |  | 0.2594 | 0.1142 | 1634 | 2.27 | 0.0232 |
| dept*trea*side*condi | AO | L | dark |  | -0.3341 | 0.1593 | 1634 | -2.1 | 0.0361 |
| depth1*region |  |  |  | 1000to2000 | -0.07844 | 0.07514 | 1634 | -1.04 | 0.2966 |
| depth1*treat*region | AO |  |  | 1000to2000 | 0.1217 | 0.1056 | 1634 | 1.15 | 0.249 |
| depth1*side*region |  | L |  | 1000to2000 | 0.1166 | 0.1033 | 1634 | 1.13 | 0.2594 |
| dept*trea*side*regio | AO | L |  | 1000to2000 | -0.1225 | 0.1389 | 1634 | -0.88 | 0.3779 |
| depth1*condit*region |  |  | dark | 1000to2000 | 0.2376 | 0.1202 | 1634 | 1.98 | 0.0482 |
| dept*trea*cond*regio | AO |  | dark | 1000to2000 | -0.3193 | 0.1805 | 1634 | -1.77 | 0.077 |
| dept*side*cond*regio |  | L | dark | 1000to2000 | -0.2067 | 0.153 | 1634 | -1.35 | 0.1769 |
| dep*tre*sid*con*regi | AO | L | dark | 1000to2000 | 0.2971 | 0.2042 | 1634 | 1.45 | 0.1459 |
| depth2 |  |  |  |  | -0.2573 | 0.1813 | 1634 | -1.42 | 0.156 |
| depth2*treat | AO |  |  |  | 0.4928 | 0.2422 | 1634 | 2.03 | 0.0421 |
| depth2*side |  | L |  |  | 0.5192 | 0.2241 | 1634 | 2.32 | 0.0206 |
| depth2*treat*side | AO | L |  |  | -0.5877 | 0.2945 | 1634 | -2 | 0.0462 |
| depth2*condition |  |  | dark |  | 0.8702 | 0.2848 | 1634 | 3.06 | 0.0023 |
| depth2*treat*conditi | AO |  | dark |  | -1.1063 | 0.4196 | 1634 | -2.64 | 0.0084 |
| depth2*side*conditio |  | L | dark |  | -0.8247 | 0.3332 | 1634 | -2.48 | 0.0134 |
| dept*trea*side*condi | AO | L | dark |  | 1.0645 | 0.4648 | 1634 | 2.29 | 0.0222 |
| depth2*region |  |  |  | 1000to2000 | 0.254 | 0.215 | 1634 | 1.18 | 0.2377 |
| depth2*treat*region | AO |  |  | 1000to2000 | -0.4129 | 0.303 | 1634 | -1.36 | 0.1732 |
| depth2*side*region |  | L |  | 1000to2000 | -0.4337 | 0.2979 | 1634 | -1.46 | 0.1456 |
| dept*trea*side*regio | AO | L |  | 1000to2000 | 0.4491 | 0.4011 | 1634 | 1.12 | 0.263 |
| depth2*condit*region |  |  | dark | 1000to2000 | -0.7536 | 0.3492 | 1634 | -2.16 | 0.0311 |
| dept*trea*cond*regio | AO |  | dark | 1000to2000 | 1.0211 | 0.5244 | 1634 | 1.95 | 0.0517 |
| dept*side*cond*regio |  | L | dark | 1000to2000 | 0.7037 | 0.4436 | 1634 | 1.59 | 0.1129 |
| dep*tre*sid*con*regi | AO | L | dark | 1000to2000 | -0.9658 | 0.5913 | 1634 | -1.63 | 0.1026 |
| depth3 |  |  |  |  | 0.4058 | 0.2071 | 1634 | 1.96 | 0.0502 |
| depth3*treat | AO |  |  |  | -0.7498 | 0.274 | 1634 | -2.74 | 0.0063 |
| depth3*side |  | L |  |  | -0.7171 | 0.2589 | 1634 | -2.77 | 0.0057 |
| depth3*treat*side | AO | L |  |  | 0.8178 | 0.3392 | 1634 | 2.41 | 0.016 |
| depth3*condition |  |  | dark |  | -1.1523 | 0.3271 | 1634 | -3.52 | 0.0004 |
| depth3*treat*conditi | AO |  | dark |  | 1.6176 | 0.4821 | 1634 | 3.36 | 0.0008 |
| depth3*side*conditio |  | L | dark |  | 1.115 | 0.3846 | 1634 | 2.9 | 0.0038 |
| dept*trea*side*condi | AO | L | dark |  | -1.4803 | 0.5396 | 1634 | -2.74 | 0.0061 |
| depth3*region |  |  |  | 1000to2000 | -0.3551 | 0.2433 | 1634 | -1.46 | 0.1446 |
| depth3*treat*region | AO |  |  | 1000to2000 | 0.5855 | 0.3427 | 1634 | 1.71 | 0.0878 |
| depth3*side*region |  | L |  | 1000to2000 | 0.6658 | 0.342 | 1634 | 1.95 | 0.0517 |
| dept*trea*side*regio | AO | L |  | 1000to2000 | -0.6719 | 0.4623 | 1634 | -1.45 | 0.1463 |
| depth3*condit*region |  |  | dark | 1000to2000 | 0.9845 | 0.3998 | 1634 | 2.46 | 0.0139 |
| dept*trea*cond*regio | AO |  | dark | 1000to2000 | -1.4227 | 0.5993 | 1634 | -2.37 | 0.0177 |
| dept*side*cond*regio |  | L | dark | 1000to2000 | -1.014 | 0.5082 | 1634 | -2 | 0.0462 |
| dep*tre*sid*con*regi | AO | L | dark | 1000to2000 | 1.3367 | 0.6828 | 1634 | 1.96 | 0.0504 |
| depth4 |  |  |  |  | -0.3911 | 0.1601 | 1648 | -2.44 | 0.0147 |
| depth4*treat | AO |  |  |  | 0.8684 | 0.2095 | 1648 | 4.15 | <.0001 |
| depth4*side |  | L |  |  | 0.7329 | 0.2091 | 1639 | 3.5 | 0.0005 |
| depth4*treat*side | AO | L |  |  | -0.8492 | 0.275 | 1639 | -3.09 | 0.002 |
| depth4*condition |  |  | dark |  | 1.1977 | 0.2492 | 1648 | 4.81 | <.0001 |
| depth4*treat*conditi | AO |  | dark |  | -1.9245 | 0.3671 | 1648 | -5.24 | <.0001 |
| depth4*side*conditio |  | L | dark |  | -1.2195 | 0.3032 | 1639 | -4.02 | <.0001 |
| dept*trea*side*condi | AO | L | dark |  | 1.709 | 0.4348 | 1639 | 3.93 | <.0001 |
| depth4*region |  |  |  | 1000to2000 | 0.3537 | 0.1992 | 1645 | 1.78 | 0.076 |
| depth4*treat*region | AO |  |  | 1000to2000 | -0.6856 | 0.2752 | 1645 | -2.49 | 0.0128 |
| depth4*side*region |  | L |  | 1000to2000 | -0.7518 | 0.2825 | 1634 | -2.66 | 0.0079 |
| dept*trea*side*regio | AO | L |  | 1000to2000 | 0.7707 | 0.3828 | 1634 | 2.01 | 0.0442 |
| depth4*condit*region |  |  | dark | 1000to2000 | -1.0952 | 0.3136 | 1645 | -3.49 | 0.0005 |
| dept*trea*cond*regio | AO |  | dark | 1000to2000 | 1.7046 | 0.4647 | 1645 | 3.67 | 0.0003 |
| dept*side*cond*regio |  | L | dark | 1000to2000 | 1.2085 | 0.404 | 1634 | 2.99 | 0.0028 |
| dep*tre*sid*con*regi | AO | L | dark | 1000to2000 | -1.5695 | 0.5624 | 1634 | -2.79 | 0.0053 |
| depth5 |  |  |  |  | 0.2467 | 0.1458 | 1634 | 1.69 | 0.0908 |
| depth5*treat | AO |  |  |  | -1.0283 | 0.1916 | 1634 | -5.37 | <.0001 |
| depth5*side |  | L |  |  | -0.8172 | 0.2008 | 1634 | -4.07 | <.0001 |
| depth5*treat*side | AO | L |  |  | 1.0034 | 0.2669 | 1634 | 3.76 | 0.0002 |
| depth5*condition |  |  | dark |  | -1.5616 | 0.213 | 1634 | -7.33 | <.0001 |
| depth5*treat*conditi | AO |  | dark |  | 2.349 | 0.3144 | 1634 | 7.47 | <.0001 |
| depth5*side*conditio |  | L | dark |  | 1.6242 | 0.277 | 1634 | 5.86 | <.0001 |
| dept*trea*side*condi | AO | L | dark |  | -2.2324 | 0.4081 | 1634 | -5.47 | <.0001 |
| depth5*region |  |  |  | 1000to2000 | -0.3257 | 0.201 | 1634 | -1.62 | 0.1053 |
| depth5*treat*region | AO |  |  | 1000to2000 | 1.0094 | 0.2706 | 1634 | 3.73 | 0.0002 |
| depth5*side*region |  | L |  | 1000to2000 | 0.929 | 0.2829 | 1634 | 3.28 | 0.001 |
| dept*trea*side*regio | AO | L |  | 1000to2000 | -1.075 | 0.381 | 1634 | -2.82 | 0.0048 |
| depth5*condit*region |  |  | dark | 1000to2000 | 1.6369 | 0.2866 | 1634 | 5.71 | <.0001 |
| dept*trea*cond*regio | AO |  | dark | 1000to2000 | -2.3702 | 0.4207 | 1634 | -5.63 | <.0001 |
| dept*side*cond*regio |  | L | dark | 1000to2000 | -1.7521 | 0.3819 | 1634 | -4.59 | <.0001 |
| dep*tre*sid*con*regi | AO | L | dark | 1000to2000 | 2.2555 | 0.5542 | 1634 | 4.07 | <.0001 |

## Table S2. Covariance parameters estimated for the model fit to 1/T1.

| Parameter | Intercept | Side | Region | Side* Region | depth4 | depth4* Side | depth4* Region | depthc | depthc* Region |
| --- | --- | --- | --- | --- | --- | --- | --- | --- | --- |
| Intercept | 0.008304 | -0.00993 | -0.00441 | 0.003455 | -0.00104 | -0.00019 | 0.001192 | 0.000149 | -0.00013 |
| Side | -0.00993 | 0.01925 | 0.002856 | -0.00355 | 0.00058 | 0.001116 | -0.00078 | -0.0001 | 0.000098 |
| Region | -0.00441 | 0.002856 | 0.005905 | -0.0056 | 0.000706 | 0.000014 | -0.00115 | -0.00012 | 0.00011 |
| Side*Region | 0.003455 | -0.00355 | -0.0056 | 0.009369 | 0.000321 | -0.00028 | 0.000568 | 0.000068 | -0.00009 |
| depth4 | -0.00104 | 0.00058 | 0.000706 | 0.000321 | 0.00051 | -0.00009 | -0.00043 | -0.00003 | 0.000018 |
| depth4*Side | -0.00019 | 0.001116 | 0.000014 | -0.00028 | -0.00009 | 0.000209 | -0.00003 | 2.52E-06 | 6.10E-07 |
| depth4*Region | 0.001192 | -0.00078 | -0.00115 | 0.000568 | -0.00043 | -0.00003 | 0.000596 | 0.000035 | -0.00003 |
| depthc | 0.000149 | -0.0001 | -0.00012 | 0.000068 | -0.00003 | 2.52E-06 | 0.000035 | 4.30E-06 | -2.87E-06 |
| depthc*Region | -0.00013 | 0.000098 | 0.00011 | -0.00009 | 0.000018 | 6.10E-07 | -0.00003 | -2.87E-06 | 4.09E-06 |
|  |  |  |  |  |  |  |  |  |  |
| Variance | **Estimate** | **Standard Error** |  |  |  |  |  |  |  |
| Residual | 0.003168 | 0.000111 |  |  |  |  |  |  |  |

## Table S3. Fixed effect parameter estimates for the model fit to ONL thickness on the inferior side.

| Effect | group | Estimate | Standard Error | DF | t Value | Pr > \|t\| |
| --- | --- | --- | --- | --- | --- | --- |
| Intercept |  | 70.8412 | 1.1614 | 128.4 | 61 | <.0001 |
| depthc |  | 0.1264 | 0.003824 | 15712 | 33.06 | <.0001 |
| group | CD | 1.07 | 1.4007 | 128.4 | 0.76 | 0.4463 |
| group | CL | 9.407 | 1.6424 | 128.4 | 5.73 | <.0001 |
| group | SDD | 17.0195 | 1.8965 | 128.4 | 8.97 | <.0001 |
| group | SDD+AO | 17.8666 | 1.8965 | 128.4 | 9.42 | <.0001 |
| group | SDL | 7.3814 | 1.5725 | 128.4 | 4.69 | <.0001 |
| group | SDL+AO | 11.0509 | 1.5725 | 128.4 | 7.03 | <.0001 |
| group | SLL | -0.7494 | 1.6424 | 128.4 | -0.46 | 0.649 |
| depthc*group | CD | -0.00859 | 0.004612 | 15712 | -1.86 | 0.0627 |
| depthc*group | CL | 0.05823 | 0.005408 | 15712 | 10.77 | <.0001 |
| depthc*group | SDD | 0.08138 | 0.006245 | 15712 | 13.03 | <.0001 |
| depthc*group | SDD+AO | 0.09594 | 0.006245 | 15712 | 15.36 | <.0001 |
| depthc*group | SDL | 0.0393 | 0.005178 | 15712 | 7.59 | <.0001 |
| depthc*group | SDL+AO | 0.06513 | 0.005178 | 15712 | 12.58 | <.0001 |
| depthc*group | SLL | 0.005594 | 0.005408 | 15712 | 1.03 | 0.301 |
| depth1 |  | -0.3613 | 0.04867 | 15712 | -7.42 | <.0001 |
| depth1*group | CD | -0.03746 | 0.0587 | 15712 | -0.64 | 0.5234 |
| depth1*group | CL | -0.3982 | 0.06883 | 15712 | -5.78 | <.0001 |
| depth1*group | SDD | -0.6739 | 0.07948 | 15712 | -8.48 | <.0001 |
| depth1*group | SDD+AO | -0.6117 | 0.07948 | 15712 | -7.7 | <.0001 |
| depth1*group | SDL | -0.2204 | 0.0659 | 15712 | -3.34 | 0.0008 |
| depth1*group | SDL+AO | -0.4889 | 0.0659 | 15712 | -7.42 | <.0001 |
| depth1*group | SLL | -0.03293 | 0.06883 | 15712 | -0.48 | 0.6324 |
| depth2 |  | 0.4463 | 0.1481 | 15712 | 3.01 | 0.0026 |
| depth2*group | CD | 0.2383 | 0.1786 | 15712 | 1.33 | 0.1821 |
| depth2*group | CL | 0.9563 | 0.2094 | 15712 | 4.57 | <.0001 |
| depth2*group | SDD | 1.5857 | 0.2418 | 15712 | 6.56 | <.0001 |
| depth2*group | SDD+AO | 1.2247 | 0.2418 | 15712 | 5.06 | <.0001 |
| depth2*group | SDL | 0.4158 | 0.2005 | 15712 | 2.07 | 0.0381 |
| depth2*group | SDL+AO | 1.1675 | 0.2005 | 15712 | 5.82 | <.0001 |
| depth2*group | SLL | 0.1541 | 0.2094 | 15712 | 0.74 | 0.4619 |
| depth3 |  | 0.2149 | 0.2041 | 15712 | 1.05 | 0.2924 |
| depth3*group | CD | -0.5801 | 0.2462 | 15712 | -2.36 | 0.0185 |
| depth3*group | CL | -0.7223 | 0.2887 | 15712 | -2.5 | 0.0124 |
| depth3*group | SDD | -1.3017 | 0.3333 | 15712 | -3.91 | <.0001 |
| depth3*group | SDD+AO | -0.6213 | 0.3333 | 15712 | -1.86 | 0.0623 |
| depth3*group | SDL | -0.2025 | 0.2764 | 15712 | -0.73 | 0.4637 |
| depth3*group | SDL+AO | -1.0166 | 0.2764 | 15712 | -3.68 | 0.0002 |
| depth3*group | SLL | -0.2622 | 0.2887 | 15712 | -0.91 | 0.3638 |
| depth4 |  | -0.4462 | 0.2122 | 15712 | -2.1 | 0.0355 |
| depth4*group | CD | 0.8313 | 0.2559 | 15712 | 3.25 | 0.0012 |
| depth4*group | CL | -0.05044 | 0.3001 | 15712 | -0.17 | 0.8665 |
| depth4*group | SDD | 0.5853 | 0.3465 | 15712 | 1.69 | 0.0912 |
| depth4*group | SDD+AO | -0.03545 | 0.3465 | 15712 | -0.1 | 0.9185 |
| depth4*group | SDL | 0.07268 | 0.2873 | 15712 | 0.25 | 0.8003 |
| depth4*group | SDL+AO | 0.5931 | 0.2873 | 15712 | 2.06 | 0.039 |
| depth4*group | SLL | 0.09977 | 0.3001 | 15712 | 0.33 | 0.7395 |
| depth5 |  | 0.2167 | 0.2334 | 624.2 | 0.93 | 0.3534 |
| depth5*group | CD | -0.8636 | 0.2814 | 624.2 | -3.07 | 0.0022 |
| depth5*group | CL | 0.6065 | 0.33 | 624.2 | 1.84 | 0.0666 |
| depth5*group | SDD | -0.08835 | 0.3811 | 624.2 | -0.23 | 0.8167 |
| depth5*group | SDD+AO | 0.294 | 0.3811 | 624.2 | 0.77 | 0.4408 |
| depth5*group | SDL | -0.02969 | 0.316 | 624.2 | -0.09 | 0.9252 |
| depth5*group | SDL+AO | -0.4127 | 0.316 | 624.2 | -1.31 | 0.192 |
| depth5*group | SLL | 0.1956 | 0.33 | 624.2 | 0.59 | 0.5536 |

## Table S4. Covariance parameters estimated for ONL thickness on the inferior side.

| Parameter | Intercept | depth5 |
| --- | --- | --- |
| Intercept | 3.5582 | -0.4105 |
| depth5 | -0.4105 | 0.06396 |
|  |  |  |
| Variance | **Estimate** | **Standard Error** |
| Residual | 3.0623 | 0.03455 |

## Table S5. Fixed effect parameter estimates for the model fit to ONL thickness on the superior side.

| Effect | group | Estimate | Standard Error | DF | t Value | Pr > \|t\| |
| --- | --- | --- | --- | --- | --- | --- |
| Intercept |  | 75.4937 | 1.8375 | 267.6 | 41.09 | <.0001 |
| depthc |  | 0.1927 | 0.006762 | 15608 | 28.5 | <.0001 |
| group | CD | -8.9979 | 2.216 | 267.6 | -4.06 | <.0001 |
| group | CL | 3.2604 | 2.598 | 267.6 | 1.25 | 0.2106 |
| group | SDD | 13.4063 | 2.9998 | 267.6 | 4.47 | <.0001 |
| group | SDD+AO | 8.8415 | 2.9999 | 267.6 | 2.95 | 0.0035 |
| group | SDL | 6.4158 | 2.4876 | 267.6 | 2.58 | 0.0104 |
| group | SDL+AO | 4.9446 | 2.4875 | 267.6 | 1.99 | 0.0479 |
| group | SLL | -4.3182 | 2.598 | 267.6 | -1.66 | 0.0977 |
| depthc*group | CD | -0.03286 | 0.008155 | 15608 | -4.03 | <.0001 |
| depthc*group | CL | -0.0162 | 0.00956 | 15608 | -1.7 | 0.0901 |
| depthc*group | SDD | 0.002225 | 0.01104 | 15608 | 0.2 | 0.8402 |
| depthc*group | SDD+AO | -0.01405 | 0.01104 | 15608 | -1.27 | 0.2032 |
| depthc*group | SDL | -0.00539 | 0.009154 | 15608 | -0.59 | 0.5561 |
| depthc*group | SDL+AO | -0.02015 | 0.009153 | 15608 | -2.2 | 0.0278 |
| depthc*group | SLL | -0.03862 | 0.00956 | 15608 | -4.04 | <.0001 |
| depth1 |  | 0.02535 | 0.1556 | 15608 | 0.16 | 0.8705 |
| depth1*group | CD | 0.5241 | 0.1876 | 15608 | 2.79 | 0.0052 |
| depth1*group | CL | -0.1772 | 0.2199 | 15608 | -0.81 | 0.4203 |
| depth1*group | SDD | -1.4913 | 0.2539 | 15608 | -5.87 | <.0001 |
| depth1*group | SDD+AO | -1.225 | 0.2539 | 15608 | -4.82 | <.0001 |
| depth1*group | SDL | -0.6879 | 0.2106 | 15608 | -3.27 | 0.0011 |
| depth1*group | SDL+AO | -0.7049 | 0.2106 | 15608 | -3.35 | 0.0008 |
| depth1*group | SLL | 0.2406 | 0.2199 | 15608 | 1.09 | 0.2739 |
| depth2 |  | -1.768 | 0.4862 | 15608 | -3.64 | 0.0003 |
| depth2*group | CD | -0.8172 | 0.5863 | 15608 | -1.39 | 0.1634 |
| depth2*group | CL | 0.5007 | 0.6873 | 15608 | 0.73 | 0.4663 |
| depth2*group | SDD | 3.8986 | 0.7935 | 15608 | 4.91 | <.0001 |
| depth2*group | SDD+AO | 3.6954 | 0.7936 | 15608 | 4.66 | <.0001 |
| depth2*group | SDL | 1.9873 | 0.6581 | 15608 | 3.02 | 0.0025 |
| depth2*group | SDL+AO | 2.2258 | 0.6581 | 15608 | 3.38 | 0.0007 |
| depth2*group | SLL | -0.5094 | 0.6873 | 15608 | -0.74 | 0.4586 |
| depth3 |  | 3.4308 | 0.6778 | 15608 | 5.06 | <.0001 |
| depth3*group | CD | -0.4054 | 0.8174 | 15608 | -0.5 | 0.6199 |
| depth3*group | CL | -0.6157 | 0.9582 | 15608 | -0.64 | 0.5205 |
| depth3*group | SDD | -2.8678 | 1.1063 | 15608 | -2.59 | 0.0095 |
| depth3*group | SDD+AO | -3.7734 | 1.1064 | 15608 | -3.41 | 0.0007 |
| depth3*group | SDL | -1.8348 | 0.9175 | 15608 | -2 | 0.0455 |
| depth3*group | SDL+AO | -2.3265 | 0.9174 | 15608 | -2.54 | 0.0112 |
| depth3*group | SLL | 0.2389 | 0.9581 | 15608 | 0.25 | 0.8031 |
| depth4 |  | -2.1347 | 0.7158 | 15608 | -2.98 | 0.0029 |
| depth4*group | CD | 1.4338 | 0.8632 | 15608 | 1.66 | 0.0968 |
| depth4*group | CL | 0.8793 | 1.012 | 15608 | 0.87 | 0.3849 |
| depth4*group | SDD | 0.4862 | 1.1685 | 15608 | 0.42 | 0.6774 |
| depth4*group | SDD+AO | 1.8303 | 1.1686 | 15608 | 1.57 | 0.1173 |
| depth4*group | SDL | 0.6465 | 0.9691 | 15608 | 0.67 | 0.5047 |
| depth4*group | SDL+AO | 0.9803 | 0.969 | 15608 | 1.01 | 0.3117 |
| depth4*group | SLL | 0.4845 | 1.012 | 15608 | 0.48 | 0.6321 |
| depth5 |  | 0.9249 | 0.7286 | 15608 | 1.27 | 0.2043 |
| depth5*group | CD | -1.1546 | 0.8787 | 15608 | -1.31 | 0.1889 |
| depth5*group | CL | -1.128 | 1.0303 | 15608 | -1.09 | 0.2736 |
| depth5*group | SDD | -0.8409 | 1.1896 | 15608 | -0.71 | 0.4797 |
| depth5*group | SDD+AO | -0.9846 | 1.1897 | 15608 | -0.83 | 0.4079 |
| depth5*group | SDL | -0.3284 | 0.9865 | 15608 | -0.33 | 0.7392 |
| depth5*group | SDL+AO | -0.3677 | 0.9864 | 15608 | -0.37 | 0.7093 |
| depth5*group | SLL | -0.9267 | 1.0303 | 15608 | -0.9 | 0.3684 |
| depth6 |  | -0.5038 | 0.7155 | 15608 | -0.7 | 0.4814 |
| depth6*group | CD | 0.3147 | 0.8629 | 15608 | 0.36 | 0.7154 |
| depth6*group | CL | 0.584 | 1.0118 | 15608 | 0.58 | 0.5638 |
| depth6*group | SDD | 1.6318 | 1.1683 | 15608 | 1.4 | 0.1625 |
| depth6*group | SDD+AO | 0.5118 | 1.1684 | 15608 | 0.44 | 0.6613 |
| depth6*group | SDL | 0.335 | 0.9688 | 15608 | 0.35 | 0.7295 |
| depth6*group | SDL+AO | 0.361 | 0.9687 | 15608 | 0.37 | 0.7094 |
| depth6*group | SLL | 0.2652 | 1.0118 | 15608 | 0.26 | 0.7932 |
| depth7 |  | -0.00325 | 0.7174 | 2530 | 0 | 0.9964 |
| depth7*group | CD | 0.3882 | 0.8652 | 2530 | 0.45 | 0.6537 |
| depth7*group | CL | 0.3727 | 1.0146 | 2530 | 0.37 | 0.7134 |
| depth7*group | SDD | -1.6989 | 1.1715 | 2530 | -1.45 | 0.1471 |
| depth7*group | SDD+AO | -0.2071 | 1.1715 | 2530 | -0.18 | 0.8597 |
| depth7*group | SDL | -0.3586 | 0.9714 | 2530 | -0.37 | 0.712 |
| depth7*group | SDL+AO | -0.4164 | 0.9714 | 2530 | -0.43 | 0.6682 |
| depth7*group | SLL | 0.6644 | 1.0146 | 2530 | 0.65 | 0.5126 |

## Table S6. Covariance parameters estimated for ONL thickness on the superior side.

| Parameter | Intercept | depth7 |
| --- | --- | --- |
| Intercept | 6.1505 | -1.073 |
| depth7 | -1.073 | 0.28 |
|  |  |  |
| Variance | **Estimate** | **Standard Error** |
| Residual | 4.4913 | 0.05084 |

## Table S7. Fixed effect parameter estimates for the model fit to ELM-RPE thickness on the inferior side.

| Effect | group | Estimate | Standard Error | DF | t Value | Pr > \|t\| |
| --- | --- | --- | --- | --- | --- | --- |
| Intercept |  | 53.9982 | 0.9106 | 153.8 | 59.3 | <.0001 |
| depthc |  | 0.02639 | 0.003009 | 15696 | 8.77 | <.0001 |
| group | CD | -3.322 | 1.0983 | 153.8 | -3.02 | 0.0029 |
| group | CL | 0.9629 | 1.2878 | 153.8 | 0.75 | 0.4558 |
| group | SDD | -2.1432 | 1.4871 | 153.8 | -1.44 | 0.1515 |
| group | SDD+AO | -4.2468 | 1.4871 | 153.8 | -2.86 | 0.0049 |
| group | SDL | 2.1965 | 1.233 | 153.8 | 1.78 | 0.0768 |
| group | SDL+AO | 4.583 | 1.233 | 153.8 | 3.72 | 0.0003 |
| group | SLL | 1.4516 | 1.2878 | 153.8 | 1.13 | 0.2614 |
| depthc*group | CD | -0.01372 | 0.003629 | 15696 | -3.78 | 0.0002 |
| depthc*group | CL | -0.01548 | 0.004255 | 15696 | -3.64 | 0.0003 |
| depthc*group | SDD | 0.001244 | 0.004913 | 15696 | 0.25 | 0.8002 |
| depthc*group | SDD+AO | -0.00779 | 0.004913 | 15696 | -1.58 | 0.1131 |
| depthc*group | SDL | 0.001982 | 0.004074 | 15696 | 0.49 | 0.6265 |
| depthc*group | SDL+AO | 0.00733 | 0.004074 | 15696 | 1.8 | 0.072 |
| depthc*group | SLL | 0.009894 | 0.004255 | 15696 | 2.33 | 0.0201 |
| depth1 |  | -0.1013 | 0.07034 | 15696 | -1.44 | 0.1499 |
| depth1*group | CD | 0.08997 | 0.08483 | 15696 | 1.06 | 0.2889 |
| depth1*group | CL | 0.4036 | 0.09948 | 15696 | 4.06 | <.0001 |
| depth1*group | SDD | 0.08053 | 0.1149 | 15696 | 0.7 | 0.4833 |
| depth1*group | SDD+AO | 0.1097 | 0.1149 | 15696 | 0.96 | 0.3396 |
| depth1*group | SDL | 0.1503 | 0.09524 | 15696 | 1.58 | 0.1145 |
| depth1*group | SDL+AO | 0.03867 | 0.09524 | 15696 | 0.41 | 0.6847 |
| depth1*group | SLL | 0.03689 | 0.09947 | 15696 | 0.37 | 0.7108 |
| depth2 |  | 0.1048 | 0.2144 | 15696 | 0.49 | 0.6248 |
| depth2*group | CD | 0.009943 | 0.2585 | 15696 | 0.04 | 0.9693 |
| depth2*group | CL | -0.8974 | 0.3032 | 15696 | -2.96 | 0.0031 |
| depth2*group | SDD | -0.02369 | 0.3501 | 15696 | -0.07 | 0.946 |
| depth2*group | SDD+AO | -0.09475 | 0.3501 | 15696 | -0.27 | 0.7867 |
| depth2*group | SDL | -0.5986 | 0.2903 | 15696 | -2.06 | 0.0392 |
| depth2*group | SDL+AO | -0.05607 | 0.2903 | 15696 | -0.19 | 0.8468 |
| depth2*group | SLL | -0.1567 | 0.3032 | 15696 | -0.52 | 0.6052 |
| depth3 |  | 0.02832 | 0.2996 | 15696 | 0.09 | 0.9247 |
| depth3*group | CD | -0.4987 | 0.3613 | 15696 | -1.38 | 0.1675 |
| depth3*group | CL | 0.425 | 0.4237 | 15696 | 1 | 0.3159 |
| depth3*group | SDD | -0.2848 | 0.4893 | 15696 | -0.58 | 0.5605 |
| depth3*group | SDD+AO | -0.09897 | 0.4893 | 15696 | -0.2 | 0.8397 |
| depth3*group | SDL | 1.0547 | 0.4057 | 15696 | 2.6 | 0.0093 |
| depth3*group | SDL+AO | -0.2035 | 0.4057 | 15696 | -0.5 | 0.6159 |
| depth3*group | SLL | 0.2306 | 0.4237 | 15696 | 0.54 | 0.5863 |
| depth4 |  | -0.0071 | 0.3221 | 15696 | -0.02 | 0.9824 |
| depth4*group | CD | 0.9184 | 0.3884 | 15696 | 2.36 | 0.0181 |
| depth4*group | CL | 0.004874 | 0.4555 | 15696 | 0.01 | 0.9915 |
| depth4*group | SDD | 0.3784 | 0.5259 | 15696 | 0.72 | 0.4719 |
| depth4*group | SDD+AO | -0.1247 | 0.5259 | 15696 | -0.24 | 0.8126 |
| depth4*group | SDL | -1.1134 | 0.4361 | 15696 | -2.55 | 0.0107 |
| depth4*group | SDL+AO | 0.5719 | 0.4361 | 15696 | 1.31 | 0.1897 |
| depth4*group | SLL | -0.2557 | 0.4555 | 15696 | -0.56 | 0.5746 |
| depth5 |  | 0.3098 | 0.3292 | 15696 | 0.94 | 0.3466 |
| depth5*group | CD | -1.3313 | 0.397 | 15696 | -3.35 | 0.0008 |
| depth5*group | CL | 0.3393 | 0.4655 | 15696 | 0.73 | 0.4661 |
| depth5*group | SDD | -0.7529 | 0.5375 | 15696 | -1.4 | 0.1613 |
| depth5*group | SDD+AO | -0.00907 | 0.5375 | 15696 | -0.02 | 0.9865 |
| depth5*group | SDL | 0.3352 | 0.4457 | 15696 | 0.75 | 0.452 |
| depth5*group | SDL+AO | -0.9748 | 0.4457 | 15696 | -2.19 | 0.0287 |
| depth5*group | SLL | 0.05813 | 0.4655 | 15696 | 0.12 | 0.9006 |
| depth6 |  | -1.0274 | 0.3221 | 15696 | -3.19 | 0.0014 |
| depth6*group | CD | 1.6139 | 0.3884 | 15696 | 4.16 | <.0001 |
| depth6*group | CL | -0.01596 | 0.4555 | 15696 | -0.04 | 0.972 |
| depth6*group | SDD | 1.6337 | 0.5259 | 15696 | 3.11 | 0.0019 |
| depth6*group | SDD+AO | 0.9741 | 0.5259 | 15696 | 1.85 | 0.064 |
| depth6*group | SDL | 1.0679 | 0.4361 | 15696 | 2.45 | 0.0143 |
| depth6*group | SDL+AO | 1.6387 | 0.4361 | 15696 | 3.76 | 0.0002 |
| depth6*group | SLL | 0.6751 | 0.4555 | 15696 | 1.48 | 0.1383 |
| depth7 |  | 1.2404 | 0.3139 | 3495 | 3.95 | <.0001 |
| depth7*group | CD | -1.0686 | 0.3786 | 3495 | -2.82 | 0.0048 |
| depth7*group | CL | -0.7541 | 0.444 | 3495 | -1.7 | 0.0895 |
| depth7*group | SDD | -1.8925 | 0.5126 | 3495 | -3.69 | 0.0002 |
| depth7*group | SDD+AO | -1.3364 | 0.5126 | 3495 | -2.61 | 0.0092 |
| depth7*group | SDL | -1.6914 | 0.4251 | 3495 | -3.98 | <.0001 |
| depth7*group | SDL+AO | -1.7602 | 0.4251 | 3495 | -4.14 | <.0001 |
| depth7*group | SLL | -1.1018 | 0.444 | 3495 | -2.48 | 0.0131 |

## Table S8. Covariance parameters estimated for ELM-RPE thickness on the inferior side.

| Parameter | Intercept | depth7 |
| --- | --- | --- |
| Intercept | 2.0008 | -0.1375 |
| depth7 | -0.1375 | 0.04392 |
|  |  |  |
| Variance | **Estimate** | **Standard Error** |
| 0.9155 | 0.01033 | 0.9155 |

## Table S9. Fixed effect parameter estimates for the model fit to ELM-RPE thickness on the superior side.

| Effect | group | Estimate | Standard | DF | t Value | Pr > \|t\| |
| --- | --- | --- | --- | --- | --- | --- |
| Intercept |  | 53.6893 | 0.9909 | 146.8 | 54.18 | <.0001 |
| depthc |  | 0.02995 | 0.003213 | 15564 | 9.32 | <.0001 |
| group | CD | -4.8988 | 1.1934 | 146.8 | -4.1 | <.0001 |
| group | CL | 4.1719 | 1.4004 | 146.8 | 2.98 | 0.0034 |
| group | SDD | -5.8318 | 1.6165 | 146.8 | -3.61 | 0.0004 |
| group | SDD+AO | -12.8901 | 1.6166 | 146.8 | -7.97 | <.0001 |
| group | SDL | -2.9362 | 1.3416 | 146.8 | -2.19 | 0.0302 |
| group | SDL+AO | 0.532 | 1.3416 | 146.8 | 0.4 | 0.6923 |
| group | SLL | -2.3326 | 1.4015 | 146.8 | -1.66 | 0.0982 |
| depthc*group | CD | -0.02077 | 0.003864 | 15564 | -5.37 | <.0001 |
| depthc*group | CL | -0.0016 | 0.004538 | 15564 | -0.35 | 0.7244 |
| depthc*group | SDD | -0.02842 | 0.005237 | 15564 | -5.43 | <.0001 |
| depthc*group | SDD+AO | -0.04443 | 0.005237 | 15564 | -8.48 | <.0001 |
| depthc*group | SDL | -0.03251 | 0.00435 | 15564 | -7.47 | <.0001 |
| depthc*group | SDL+AO | -0.01836 | 0.004349 | 15564 | -4.22 | <.0001 |
| depthc*group | SLL | -0.01716 | 0.004544 | 15564 | -3.78 | 0.0002 |
| depth1 |  | -0.4084 | 0.09486 | 15564 | -4.31 | <.0001 |
| depth1*group | CD | 0.1908 | 0.114 | 15564 | 1.67 | 0.0941 |
| depth1*group | CL | 0.2032 | 0.1339 | 15564 | 1.52 | 0.1292 |
| depth1*group | SDD | 0.8324 | 0.1545 | 15564 | 5.39 | <.0001 |
| depth1*group | SDD+AO | 1.2445 | 0.1545 | 15564 | 8.06 | <.0001 |
| depth1*group | SDL | 0.5494 | 0.1284 | 15564 | 4.28 | <.0001 |
| depth1*group | SDL+AO | 0.3051 | 0.1284 | 15564 | 2.38 | 0.0175 |
| depth1*group | SLL | 0.4921 | 0.1342 | 15564 | 3.67 | 0.0002 |
| depth2 |  | 1.3288 | 0.2931 | 15564 | 4.53 | <.0001 |
| depth2*group | CD | -0.5299 | 0.352 | 15564 | -1.51 | 0.1323 |
| depth2*group | CL | -0.4054 | 0.4138 | 15564 | -0.98 | 0.3272 |
| depth2*group | SDD | -2.5507 | 0.4775 | 15564 | -5.34 | <.0001 |
| depth2*group | SDD+AO | -3.5408 | 0.4773 | 15564 | -7.42 | <.0001 |
| depth2*group | SDL | -1.4061 | 0.3968 | 15564 | -3.54 | 0.0004 |
| depth2*group | SDL+AO | -0.7218 | 0.3966 | 15564 | -1.82 | 0.0688 |
| depth2*group | SLL | -1.4569 | 0.4148 | 15564 | -3.51 | 0.0004 |
| depth3 |  | -1.9371 | 0.4141 | 15564 | -4.68 | <.0001 |
| depth3*group | CD | 1.066 | 0.4973 | 15564 | 2.14 | 0.0321 |
| depth3*group | CL | 0.04231 | 0.5848 | 15564 | 0.07 | 0.9423 |
| depth3*group | SDD | 3.277 | 0.675 | 15564 | 4.85 | <.0001 |
| depth3*group | SDD+AO | 3.8482 | 0.6743 | 15564 | 5.71 | <.0001 |
| depth3*group | SDL | 1.4545 | 0.5606 | 15564 | 2.59 | 0.0095 |
| depth3*group | SDL+AO | 0.7628 | 0.5602 | 15564 | 1.36 | 0.1733 |
| depth3*group | SLL | 1.8068 | 0.5861 | 15564 | 3.08 | 0.0021 |
| depth4 |  | 1.8458 | 0.4454 | 15564 | 4.14 | <.0001 |
| depth4*group | CD | -1.7856 | 0.5354 | 15564 | -3.34 | 0.0009 |
| depth4*group | CL | 0.0564 | 0.6295 | 15564 | 0.09 | 0.9286 |
| depth4*group | SDD | -2.6972 | 0.7268 | 15564 | -3.71 | 0.0002 |
| depth4*group | SDD+AO | -2.1892 | 0.7256 | 15564 | -3.02 | 0.0026 |
| depth4*group | SDL | -1.1772 | 0.6031 | 15564 | -1.95 | 0.051 |
| depth4*group | SDL+AO | -1.057 | 0.6025 | 15564 | -1.75 | 0.0794 |
| depth4*group | SLL | -1.7347 | 0.6304 | 15564 | -2.75 | 0.0059 |
| depth5 |  | -1.4883 | 0.4635 | 15564 | -3.21 | 0.0013 |
| depth5*group | CD | 1.7593 | 0.5579 | 15564 | 3.15 | 0.0016 |
| depth5*group | CL | 0.593 | 0.6551 | 15564 | 0.91 | 0.3654 |
| depth5*group | SDD | 1.767 | 0.7565 | 15564 | 2.34 | 0.0195 |
| depth5*group | SDD+AO | 1.089 | 0.7554 | 15564 | 1.44 | 0.1495 |
| depth5*group | SDL | 1.1111 | 0.6274 | 15564 | 1.77 | 0.0766 |
| depth5*group | SDL+AO | 1.7079 | 0.627 | 15564 | 2.72 | 0.0065 |
| depth5*group | SLL | 2.0228 | 0.6555 | 15564 | 3.09 | 0.002 |
| depth6 |  | 1.0589 | 0.4634 | 15564 | 2.28 | 0.0223 |
| depth6*group | CD | -0.9638 | 0.5583 | 15564 | -1.73 | 0.0843 |
| depth6*group | CL | -0.8605 | 0.6549 | 15564 | -1.31 | 0.1889 |
| depth6*group | SDD | -0.8069 | 0.7562 | 15564 | -1.07 | 0.286 |
| depth6*group | SDD+AO | -1.0919 | 0.7557 | 15564 | -1.44 | 0.1485 |
| depth6*group | SDL | -0.7439 | 0.6272 | 15564 | -1.19 | 0.2356 |
| depth6*group | SDL+AO | -1.8625 | 0.627 | 15564 | -2.97 | 0.003 |
| depth6*group | SLL | -1.9994 | 0.6549 | 15564 | -3.05 | 0.0023 |
| depth7 |  | -0.5559 | 0.4897 | 1089 | -1.14 | 0.2565 |
| depth7*group | CD | 0.4865 | 0.5903 | 1089 | 0.82 | 0.4101 |
| depth7*group | CL | 0.9174 | 0.6922 | 1089 | 1.33 | 0.1853 |
| depth7*group | SDD | 0.1463 | 0.7992 | 1089 | 0.18 | 0.8548 |
| depth7*group | SDD+AO | 0.9654 | 0.799 | 1089 | 1.21 | 0.2272 |
| depth7*group | SDL | 0.11 | 0.6627 | 1089 | 0.17 | 0.8682 |
| depth7*group | SDL+AO | 1.4032 | 0.6627 | 1089 | 2.12 | 0.0345 |
| depth7*group | SLL | 1.4498 | 0.6921 | 1089 | 2.09 | 0.0364 |
| depth8 |  | 0.3247 | 0.8403 | 59.68 | 0.39 | 0.7006 |
| depth8*group | CD | -0.5754 | 1.0134 | 59.68 | -0.57 | 0.5723 |
| depth8*group | CL | -1.3968 | 1.1883 | 59.68 | -1.18 | 0.2445 |
| depth8*group | SDD | 0.4414 | 1.3721 | 59.68 | 0.32 | 0.7488 |
| depth8*group | SDD+AO | -0.3901 | 1.3721 | 59.68 | -0.28 | 0.7772 |
| depth8*group | SDL | 0.1914 | 1.1377 | 59.68 | 0.17 | 0.8669 |
| depth8*group | SDL+AO | -0.7705 | 1.1377 | 59.68 | -0.68 | 0.5009 |
| depth8*group | SLL | -1.1202 | 1.1883 | 59.68 | -0.94 | 0.3496 |

## Table S10. Covariance parameters estimated for ELM-RPE thickness on the superior side.

| Parameter | Intercept | depth7 | depth8 |
| --- | --- | --- | --- |
| Intercept | 2.4191 | -0.2451 | 0.7763 |
| depth7 | -0.2451 | 0.2087 | -0.736 |
| depth8 | 0.7763 | -0.736 | 2.6773 |
|  |  |  |  |
| Variance | **Estimate** | **Standard Error** |  |
| Residual | 0.7578 | 0.00859 |  |

## Table S11. Fixed effect parameter estimates for the model fit to superoxide levels.

| Effect | treat | Estimate | Standard Error | DF | t Value | Pr > \|t\| |
| --- | --- | --- | --- | --- | --- | --- |
| Intercept |  | 425.18 | 65.1053 | 8 | 6.53 | 0.0002 |
| treat | exp | 55.9652 | 92.0679 | 8 | 0.61 | 0.5601 |

## Table S12. Covariance parameters estimated for the model fit to superoxide levels.

| Cov Parm | Subject | Estimate | Standard Error |
| --- | --- | --- | --- |
| Intercept | mouse(treat) | 20980 | 10595 |
| Intercept | batch(mouse*treat) | 1631.25 | 415.84 |
| Residual |  | 290.4 | 289.01 |

## Table S13. Fixed effect parameter estimates for the model fit to contrast sensitivity.

| Effect | treat | Estimate | Standard Error | DF | t Value | Pr > \|t\| |
| --- | --- | --- | --- | --- | --- | --- |
| Intercept |  | 1.4063 | 0.04276 | 32 | 32.89 | <.0001 |
| treat | 24hr | 4.10E-15 | 0.06047 | 32 | 0 | 1 |
| treat | 24hr+AO | 0.03954 | 0.06047 | 32 | 0.65 | 0.5179 |
| treat | SDL | 0.1901 | 0.06343 | 32 | 3 | 0.0052 |
| treat | SDL+AO | 0.3472 | 0.06343 | 32 | 5.47 | <.0001 |
| treat | SLL | 0.02774 | 0.06343 | 32 | 0.44 | 0.6648 |
| treat | SLL+AO | 3.90E-15 | 0.06047 | 32 | 0 | 1 |

## Table S14. Covariance parameters estimated for the model fit to contrast sensitivity.

| Cov Parm | Estimate | Standard Error |
| --- | --- | --- |
| Intercept | 0.004356 | 0.003125 |
| Residual | 0.01323 | 0.002996 |

## Table S15. Fixed effect parameter estimates for the model fit to spatial frequency threshold (SFT).

| Effect | treat | Estimate | Standard Error | DF | t Value | Pr > \|t\| |
| --- | --- | --- | --- | --- | --- | --- |
| Intercept |  | 0.4171 | 0.002792 | 32 | 149.41 | <.0001 |
| treat | 24hr | -0.00408 | 0.003948 | 32 | -1.03 | 0.3087 |
| treat | 24hr+AO | -0.00183 | 0.003948 | 32 | -0.46 | 0.6455 |
| treat | SDL | -0.00438 | 0.004141 | 32 | -1.06 | 0.2977 |
| treat | SDL+AO | -0.00398 | 0.004141 | 32 | -0.96 | 0.3433 |
| treat | SLL | -0.00058 | 0.004141 | 32 | -0.14 | 0.8888 |
| treat | SLL+AO | -0.00158 | 0.003948 | 32 | -0.4 | 0.691 |

## Table S16. Covariance parameters estimated for the model fit to spatial frequency threshold (SFT).

| Cov Parm | Estimate | Standard Error |
| --- | --- | --- |
| Intercept | 0 | . |
| Residual | 0.000094 | 0.000016 |

# **Supplementary Figures:**

**Figure S1. QUEST MRI showing oxidative stress localized to peripheral superior retina:** Raw and mean 1/T1 profiles approximately 1 hour post sildenafil IP in light-adapted mice given either saline (n= 4 mice, black lines) or anti-oxidants (AO, n = 5 mice, red line) in four retinal regions: A) 1000 to 2000 inferior, B) 1000 to 2000 superior, C) 400 – 1000 inferior, and D) 400 – 1000 superior. Mean profiles are estimated based on the fit model.


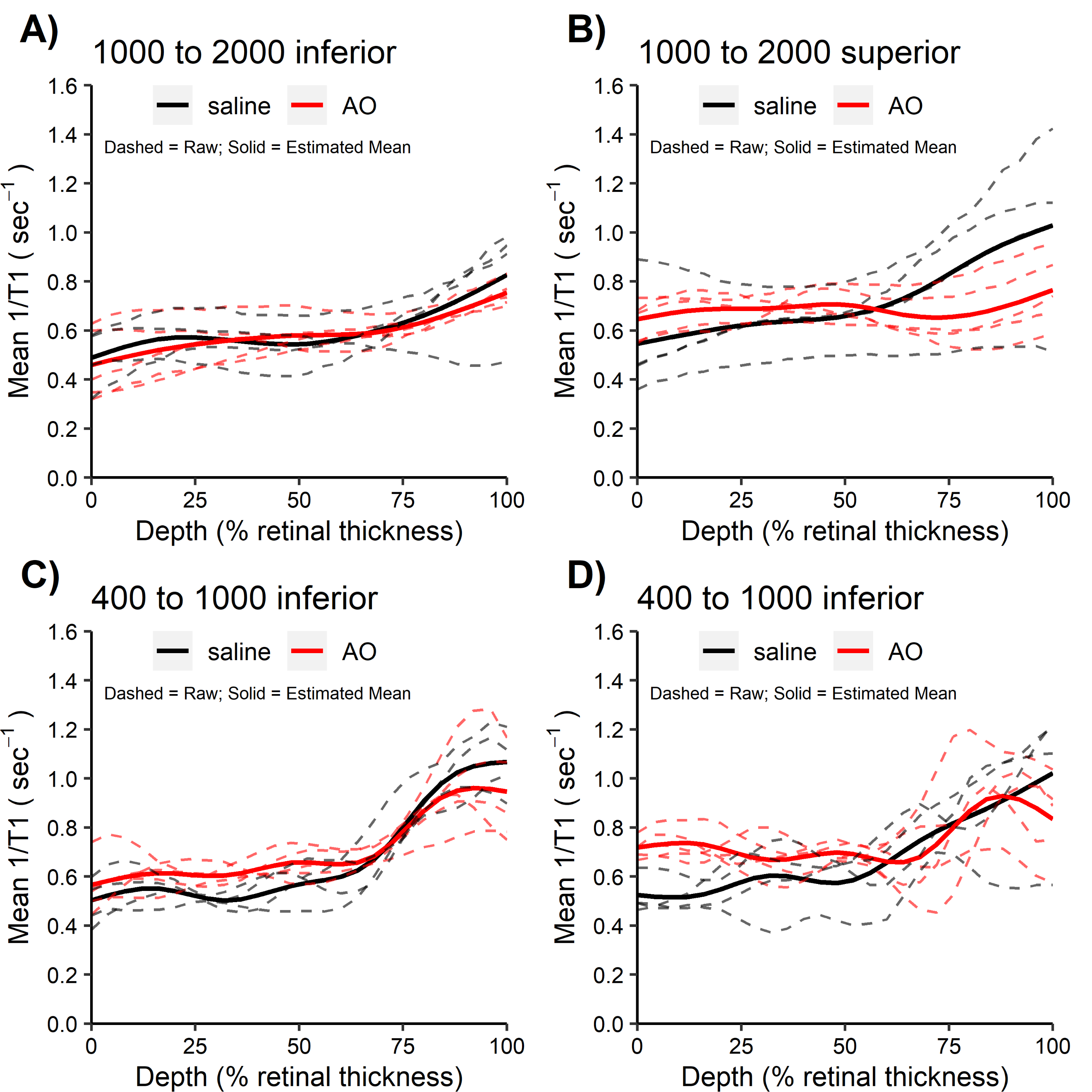


**Figure S2. Superoxide levels do not change with sildenafil treatment**. Modeled mean superoxide levels in mice treated with saline (n = 5 mice, grey bar) or treated with sildenafil (n = 5 mice, red bar). Error bars indicate 95% confidence intervals.


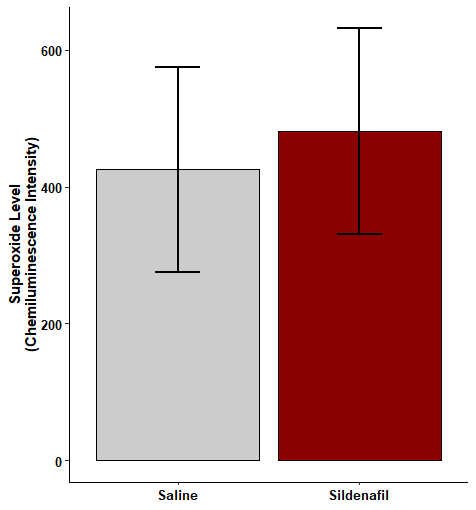


**Figure S3: 1 hr post sildenafil after 4 hr of light-adaption mice (SLL, Figure 2) shows thinner dark-like ELM-RPE.** Raw and mean profiles in two retinal regions, separated by side: A) ONL inferior retina, B) ONL superior retina, C) ELM-RPE thickness inferior retina, and D) ELM-RPE thickness superior retina in uninjected control dark (CD, n = 11 mice), control light (CL, n = 5 mice), SLL+saline (SLL, n = 5 mice), and SLL+AO (n = 5 mice). Mean profiles are estimated based on the fit model.

**Figure S4: 1 hr post sildenafil after 4 hr of light-adaption mice (SLL, Figure 2) shows thinner, dark-like ELM-RPE in extended field of view.** Modeled A) ONL inferior retina, B) ONL superior retina, C) ELM-RPE thickness inferior retina, and D) ELM-RPE thickness superior retina in uninjected control light (CL, n = 7 mice, white bar), SLL+saline (SLL, n = 4 mice, green bar), and SLL+AO (n = 4 mice, red bar) in the two different retinal regions. ONL is invariant to condition. Results based on images with the extended field of view will differ from the other results due to small changes in eye rotation as the image is shifted further to one side. Results of the extended field of view are consistent with centered analysis. Error bars indicate 95% confidence intervals. The points in each plot represent the estimated mean for each mouse based on the model. Error bars indicate 95% confidence intervals. Note the same control bars are presented in each graph to facilitate comparisons.


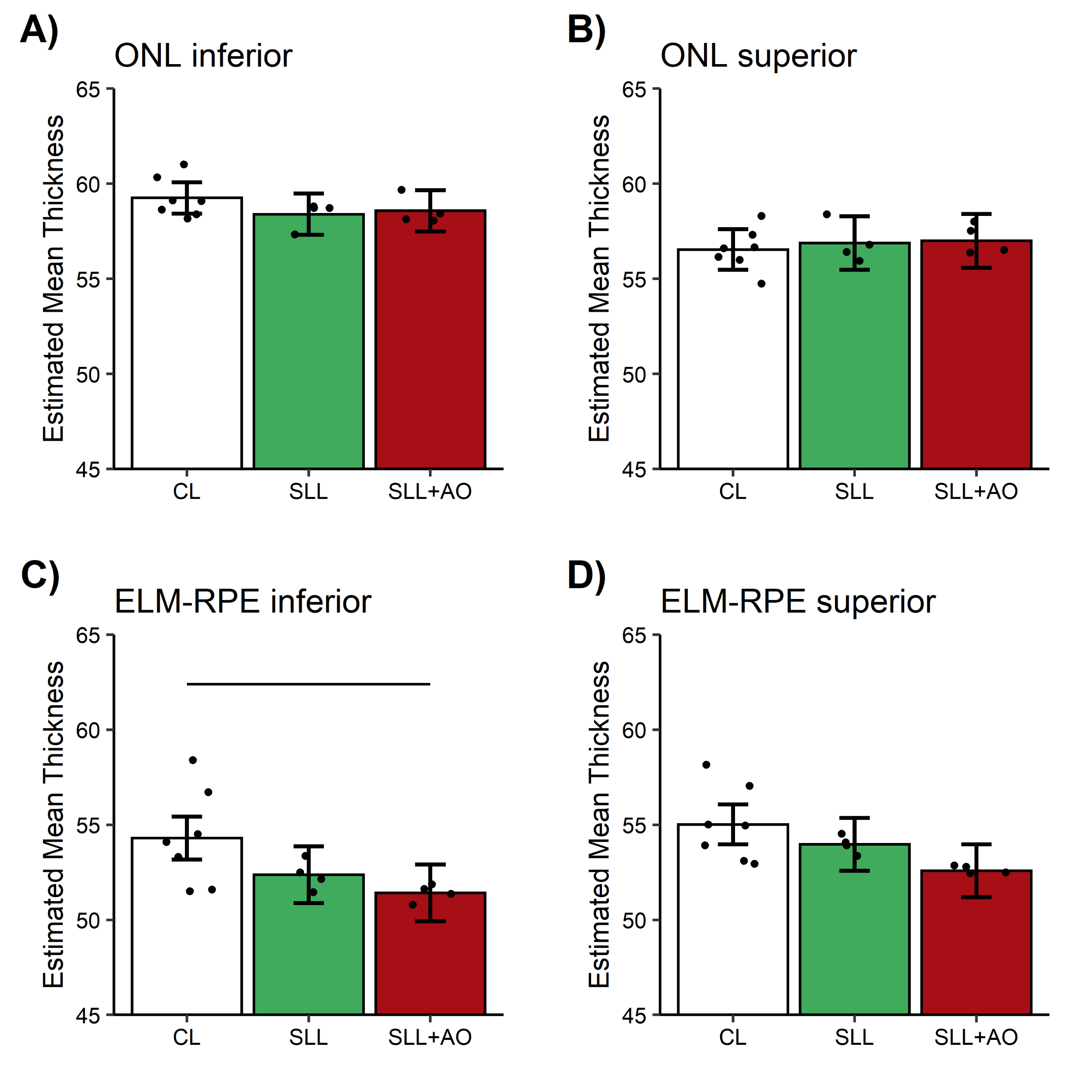


**Figure S5: 5 hr post sildenafil in light-adapted mice (SDL) shows light-like ELM-RPE.** Raw and mean OCT profiles in two retinal regions by side: A) ONL inferior retina, B) ONL superior retina, C) ELM-RPE thickness inferior retina, and D) ELM-RPE thickness superior retina in uninjected control dark (CD, n = 11 mice), control light (CL, n = 5 mice), SDL+saline (SDL, n = 6 mice), and SDL+AO (n = 6 mice). Mean profiles are estimated based on the fit model.

**Figure S6: 5 hr post sildenafil in light-adaption mice (SDL, Figure 2) shows light-like ELM-RPE in extended field of view.** Modeled A) ONL inferior retina, B) ONL superior retina, C) ELM-RPE thickness inferior retina, and D) ELM-RPE thickness superior retina in uninjected control light (CL, n = 7 mice, white bar), SDL+saline (SDL, n = 6 mice, green bar), and SDL+AO (n = 6 mice, red bar) in the two different retinal regions. The points in each plot represent the estimated mean for each mouse based on the model. Results based on images with the extended field of view will differ from the other results due to small changes in eye rotation as the image is shifted further to one side. Results of the extended field of view are consistent with centered analysis. Error bars indicate 95% confidence intervals. Note the same control bars are presented in each graph to facilitate comparisons.


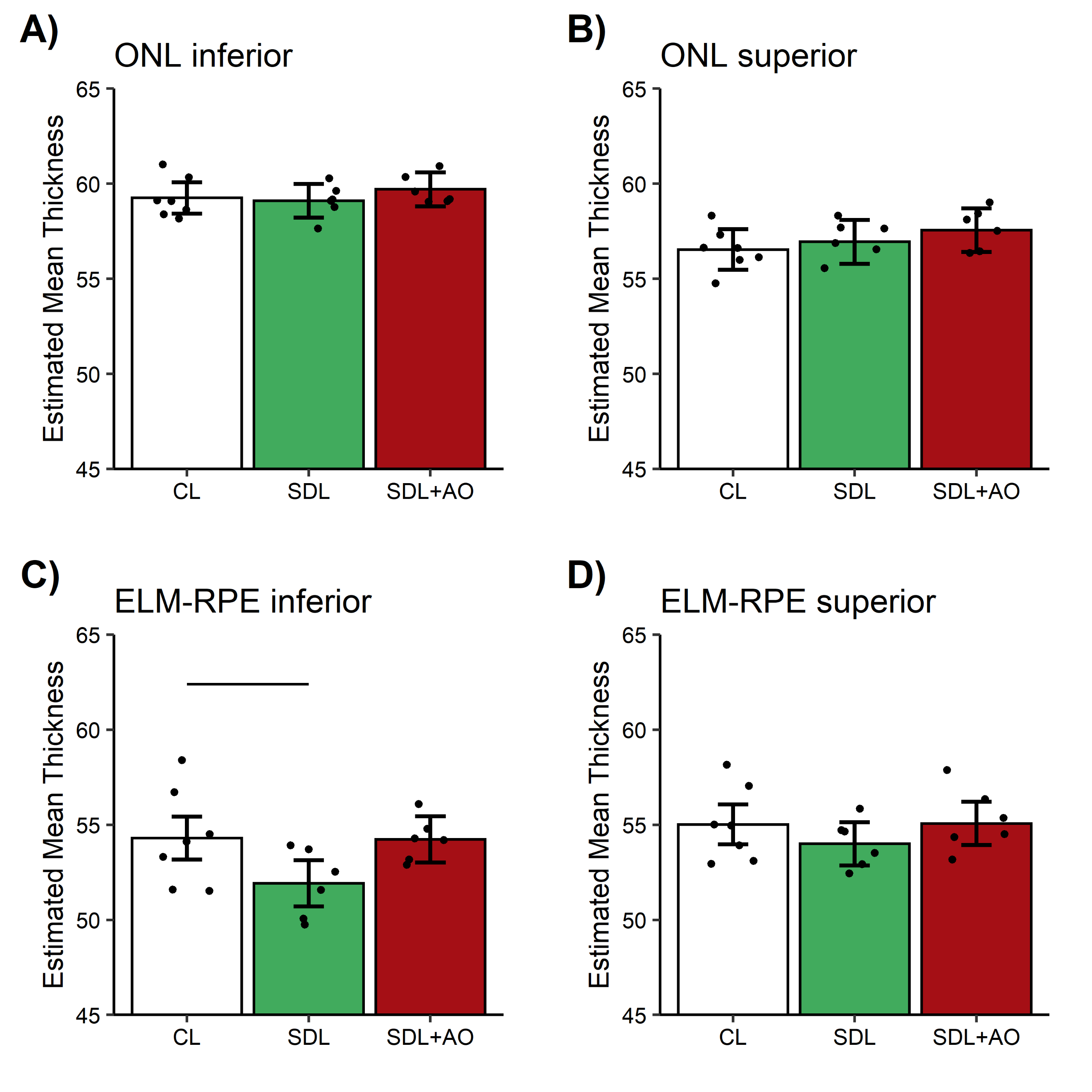


**Figure S7. QUEST MRI in dark-adapted mice do not show outer oxidative stress.** Raw and mean 1/T1 profiles approximately 1 hour post sildenafil IP in dark-adapted mice given either saline (n = 6 mice, black line) or anti-oxidants (AO, n = 3 mice, red line) in these four retinal regions: A) 1000 to 2000 inferior, B) 1000 to 2000 superior, C) 400 – 1000 inferior, and D) 400 – 1000 superior. Mean profiles are estimated based on the fit model.


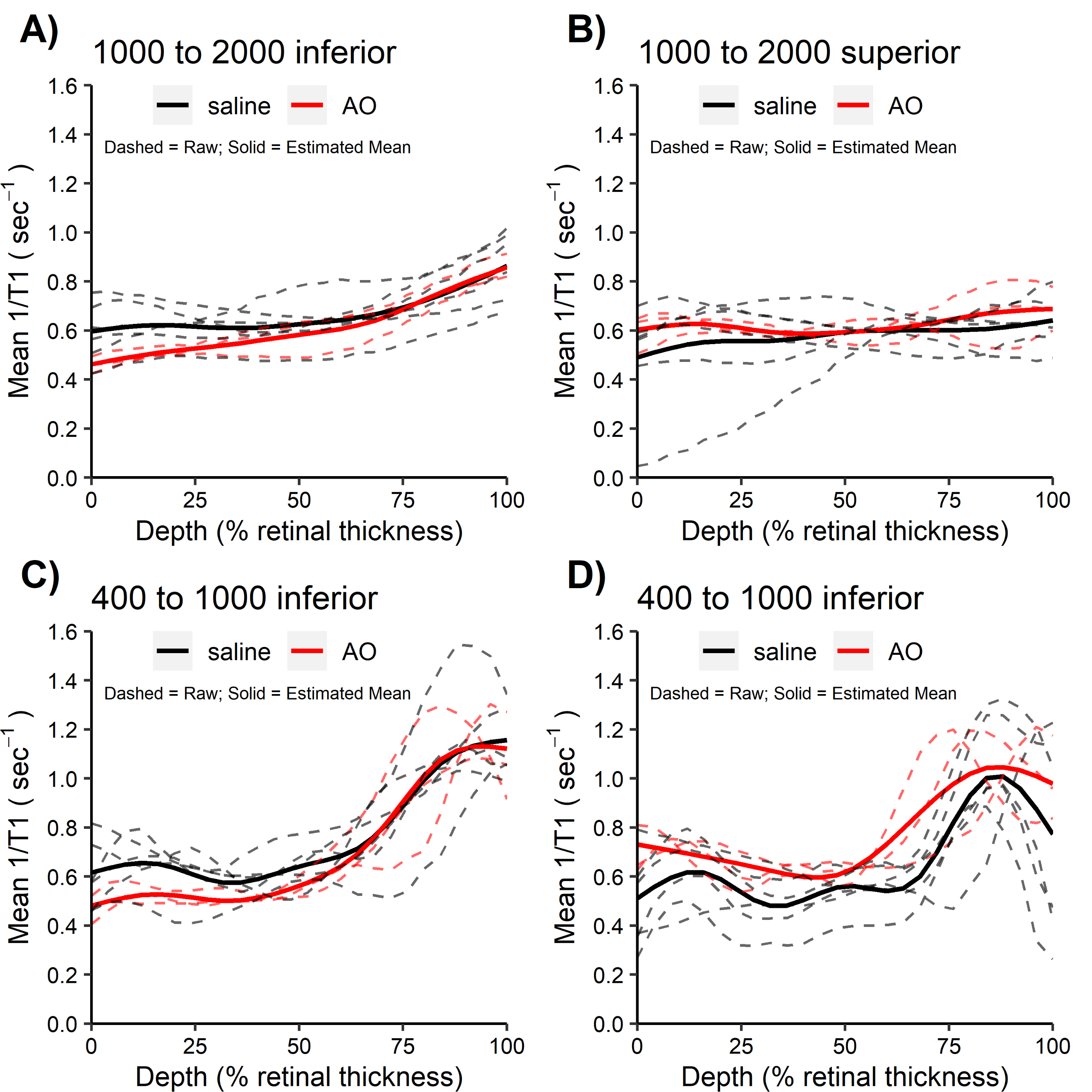


**Figure S8: 1 hr post sildenafil in dark-adapted mice shows thinner ELM-RPE.** Raw and mean OCT profiles in two retinal regions by side: A) ONL inferior retina, B) ONL superior retina, C) ELM-RPE thickness inferior retina, and D) ELM-RPE thickness superior retina in uninjected control dark (CD, n = 11 mice), control light (CL, n = 5 mice), SDD+saline (SDD, n = 3 mice), and SDD+AO (n = 3 mice). Mean profiles are estimated based on the fit model.

**Figure S9: 1 hr post sildenafil in dark-adapted mice (SDD, Figure 2) shows thinner ELM-RPE in extended field of view.** Modeled A) ONL inferior retina, B) ONL superior retina, C) ELM-RPE thickness inferior retina, and D) ELM-RPE thickness superior retina in uninjected control light (CL, n = 7 mice, white bar), SDD+saline (SDD, n = 3 mice, green bar), and SDD+AO (n = 3 mice, red bar) in the two different retinal regions. The points in each plot represent the estimated mean for each mouse based on the model. Results based on images with the extended field of view will differ from the other results due to small changes in eye rotation as the image is shifted further to one side. Results of the extended field of view are consistent with centered analysis. Error bars indicate 95% confidence intervals. Note the same control bars are presented in each graph to facilitate comparisons.


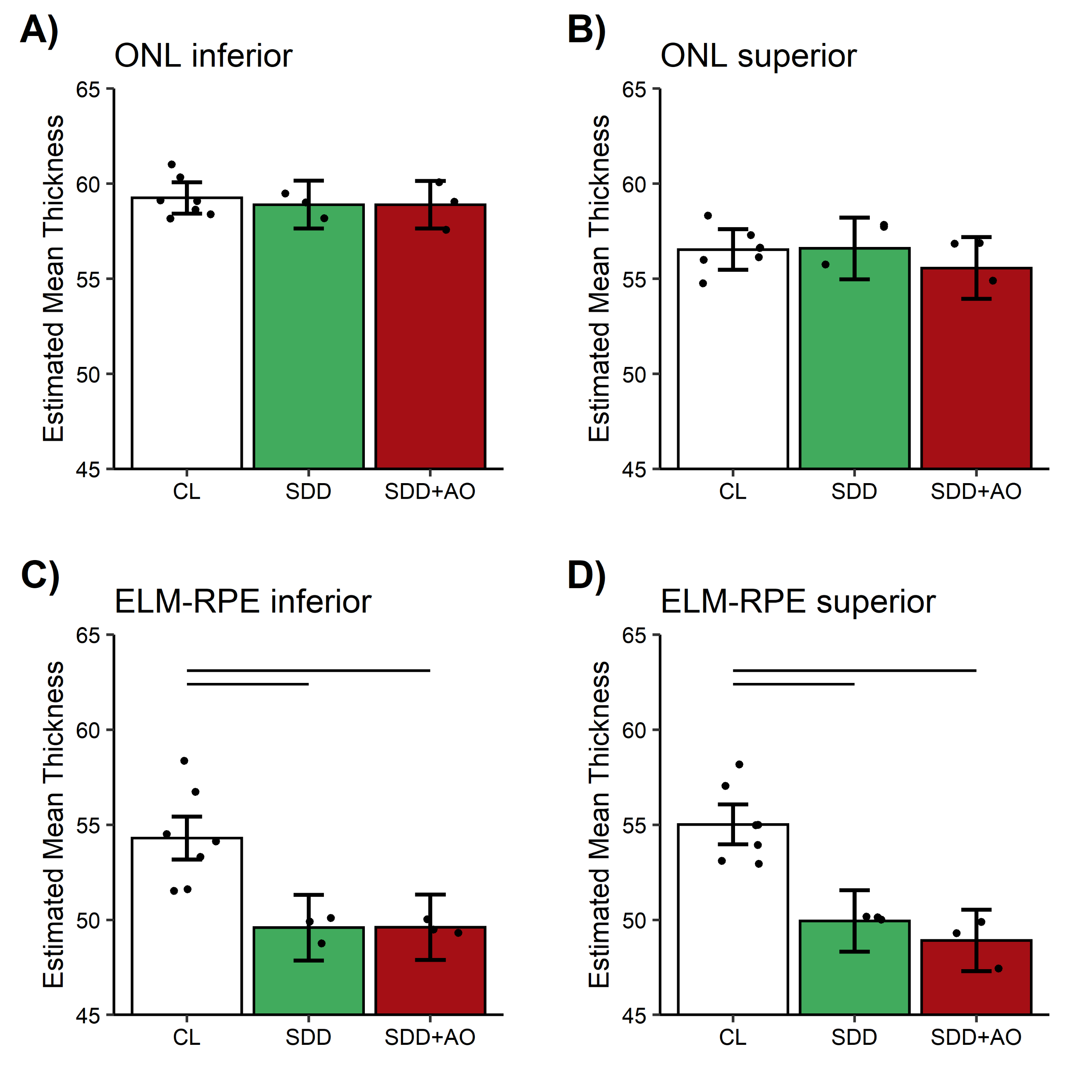

Supplement: S1 File — The supplement file contains additional details about the statistical methods used, supplemental tables, supplemental figures, and code for all analyses. (ZIP) [file pone.0245161.s001.zip › Berkowitz Sildenafil PLoS One Supplement.docx]
